# Approximate Bayesian Computation of radiocarbon and paleoenvironmental record shows population resilience on Rapa Nui (Easter Island)

**DOI:** 10.1038/s41467-021-24252-z

**Published:** 2021-06-24

**Authors:** Robert J. DiNapoli, Enrico R. Crema, Carl P. Lipo, Timothy M. Rieth, Terry L. Hunt

**Affiliations:** 1 Environmental Studies Program, Department of Anthropology, Harpur College of Arts and Sciences, Binghamton University, State University of New York, Binghamton, NY USA; 2 Department of Archaeology, University of Cambridge, Cambridge, UK; 3 International Archaeological Research Institute Inc., Honolulu, HI USA; 4 The Honors College and School of Anthropology, University of Arizona, Tucson, AZ USA

**Keywords:** Palaeoecology, Environmental social sciences, Archaeology

## Abstract

Examining how past human populations responded to environmental and climatic changes is a central focus of the historical sciences. The use of summed probability distributions (SPD) of radiocarbon dates as a proxy for estimating relative population sizes provides a widely applicable method in this research area. Paleodemographic reconstructions and modeling with SPDs, however, are stymied by a lack of accepted methods for model fitting, tools for assessing the demographic impact of environmental or climatic variables, and a means for formal multi-model comparison. These deficiencies severely limit our ability to reliably resolve crucial questions of past human-environment interactions. We propose a solution using Approximate Bayesian Computation (ABC) to fit complex demographic models to observed SPDs. Using a case study from Rapa Nui (Easter Island), a location that has long been the focus of debate regarding the impact of environmental and climatic changes on its human population, we find that past populations were resilient to environmental and climatic challenges. Our findings support a growing body of evidence showing stable and sustainable communities on the island. The ABC framework offers a novel approach for exploring regions and time periods where questions of climate-induced demographic and cultural change remain unresolved.

## Abstract (web summary)

Summed probability distributions of radiocarbon dates can be used to estimate past demography, but methods to test for associations with environmental change are lacking. Here, DiNapoli et al. propose an approach using Approximate Bayesian Computation and illustrate it in a case study of Rapa Nui.

## Introduction

Understanding how past human populations responded to ecological and climatic change continues to be a primary focus in the historical sciences, e.g.,^1–5^. While there is a growing pool of high-resolution paleoecological and paleoclimatic data, reconstructing paleodemographic patterns remains a central challenge^6–9^. Researchers have examined multiple proxies for reconstructing ancient demographic patterns^10–14^, but the use of radiocarbon frequency data is currently the most widely used approach in archeology^15–20^. Despite interpretative and methodological challenges^21–25^, the unmatched chronological resolution and the increasing availability of large collections of ^14^C dates offer unique opportunities for comparative demographic research. While there have been several methodological advances overcoming many of the early limitations of this approach, two current weaknesses remain: (1) appropriate means of formal multi-model comparison and (2) methods for directly modeling demographic effects of environmental variables. While many studies have moved away from simple visual inspections of summed probability distributions of calibrated dates (SPD) and have focused on null-hypothesis significance testing^16,17^, there is a growing interest in evaluating more complex demographic hypotheses that incorporate environmental and climatic variables. Here, we briefly review recent advances in this area of research and propose a solution based on Approximate Bayesian Computation (ABC), illustrated with a Rapa Nui (Easter Island) case study.

Several recent studies employ information criteria (IC) to formally compare contrasting demographic hypotheses against observed SPDs^26–32^. In most cases, the procedure consists of retrieving the Akaike Information Criterion (AIC) from fitted regression models where the response variable is a vector of summed probabilities and the independent variable is some transformation of the matching calendar year that emulates specific growth trajectories or in some cases incorporates additional external covariates. This approach, however, would (1) incorrectly treat the number of calendar years in the window of analyses as the sample size; (2) disregard the smearing effect of calibrated uncertainties; and (3) ignore the systematic artifacts in the SPD originating from the calibration process. As a consequence, the fitted parameters of the regression and the maximum likelihood (from which the AIC values are derived) are statistically biased and misleading (see^22,33,34^ for similar criticisms and some alternative solutions). Indeed, it is exactly for these reasons that Monte Carlo-based simulation testing was introduced nearly a decade ago in the first place^15,16^—for a given candidate hypothesis (i.e., growth model) one must compare the empirical SPD against a series of simulated theoretical curves that are drawn from the same sampling distributions and share the same measurement errors (both random and calibration-related) as the observed data. It then follows that we need similar methods that adequately account for this analytical uncertainty if robust model comparison is sought.

The challenge is further exacerbated by the growing interest in linking changes observed in SPD curves to paleoenvironmental proxies with various correlation techniques and direct comparisons, e.g.,^27,32,35–44^. While these studies offer important indications of potential associations between population and climate, there have been only limited attempts to formally model the effect of climatic or environmental changes on SPD-based population proxies, e.g.,^15,21,41,45^. Correlation tests on SPDs can suffer from similar problems as IC-based model comparison—due to the artificially large sample size inherent in evaluating annual change over durations of centuries or millennia, *p* values can be inflated leading to the appearance of overconfidence in the significance of the associations.

Furthermore, while spikes and troughs in SPD curves, or deviations from null models, may appear to be correlated with paleoenvironmental events, several non-trivial technical issues prevent simple conclusions to be drawn from ‘eye-balling’ SPDs. First, ^14^C normalization^46,47^ and back-calibration methods, used to generate simulation envelopes for model fitting^23^, can cause spurious spikes in the SPD at steep portions of the ^14^C calibration curve. In the case of ^14^C normalization, individually calibrated distributions are standardized so the posterior probability density sums to 1. In constructing SPDs, normalization inflates spikes caused by steep portions of the calibration curve, whereas non-normalized dates lack these artifacts^23,46,47^. In addition, comparing empirical SPDs to candidate models requires one to simulate distributions of radiocarbon dates consistent with the model expectations. Empirical and simulation studies have shown, however, that the choice of algorithms can produce substantially different outcomes^23,36,42^. Although recent studies have started to tackle these methodological challenges^33,34,45^, there remains a need for an inferential framework that can formalize and accommodate more complex demographic hypotheses of human–environment interaction. At the same time, we need robust techniques for model fitting and comparison that can appropriately characterize analytical uncertainty and the idiosyncratic properties of the archeological record.

**Fig. 1:**
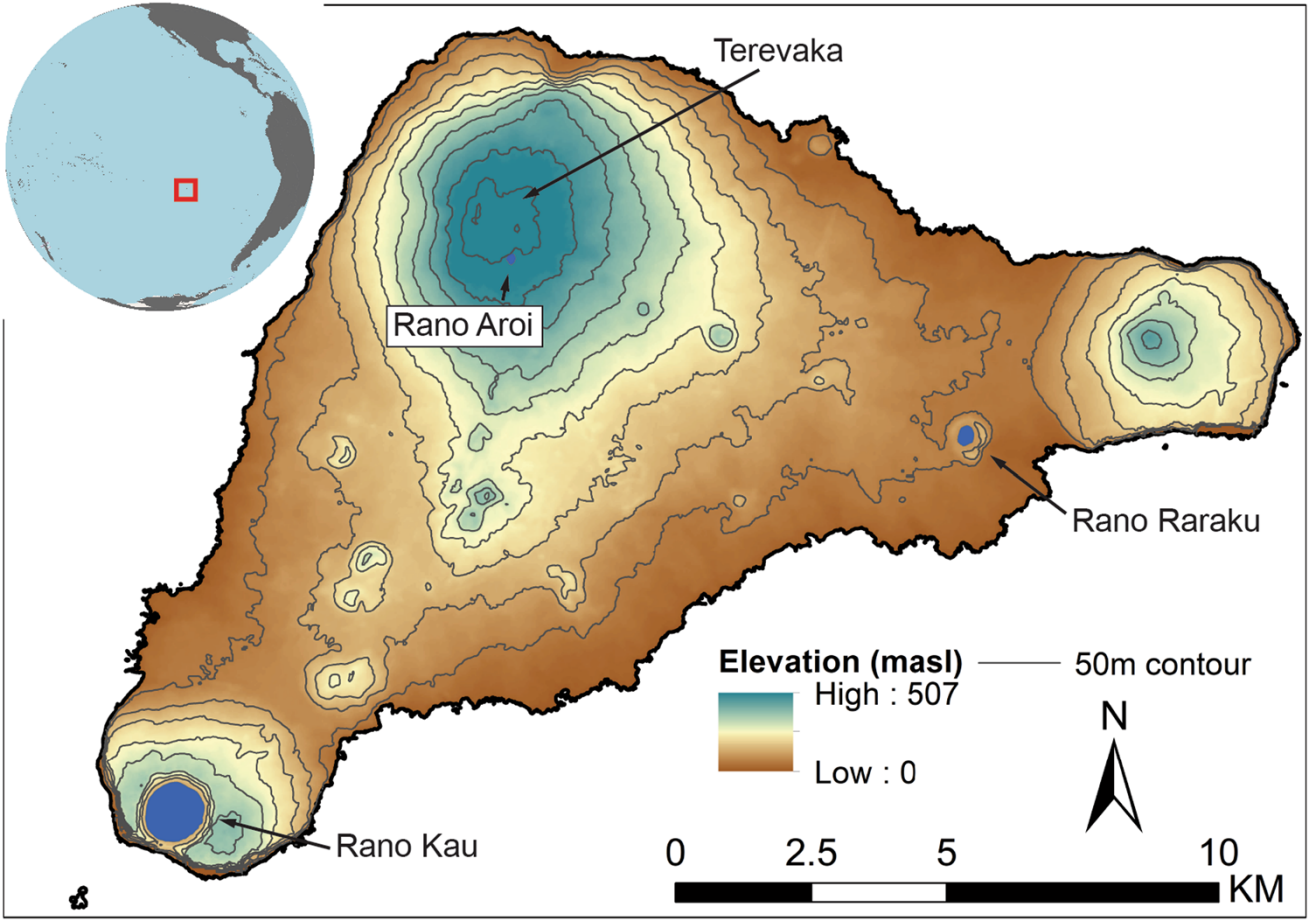
Rapa Nui (Easter Island) with the locations of places mentioned in the text. Inset shows the location of Rapa Nui in East Polynesia.

Here, we present a solution to these challenges using ABC, a flexible and powerful modeling approach originally developed in population genetics^48,49^ but recently applied in archeology^50–54^, including paleodemographic research^55,56^. We demonstrate how ABC can be used to directly integrate independent paleoenvironmental variables into demographic models and perform multi-model comparisons. To illustrate how this framework can advance paleodemographic studies, we present an analysis of Rapa Nui (Easter Island, Fig. 1), which has long been a topic of debate regarding the impact of environmental changes on the island’s pre-contact population. Our ABC approach to radiocarbon-based paleodemography can be usefully extended to other regions in the world where similar debates regarding environmentally influenced population change remain unresolved.

Rapa Nui is a small (164 km^2^) volcanic island in southeastern Polynesia (Fig. 1). Initially settled by Polynesian voyagers between the twelfth and thirteenth century AD, the island is famous for the environmental changes that followed human arrival, the remarkable achievements of Rapanui in megalithic construction, and long-standing debates surrounding demographic patterns preceding European contact^57,58^. Central to these debates is the assumption that unrestricted population growth and environmental degradation stemming from deforestation and cultivation ultimately led to a demographic and cultural collapse prior to European arrival in AD 1722^59,60^. Much of the basis for this narrative stems from the assumed incongruity of the island’s marginal environment, prolific monumental architecture, and small contact-era population size, which at the time of Dutch and Spanish visits in AD 1722 and 1770 was estimated to be no more than a few thousand^61–65^. Rapa Nui, however, was once covered in an extensive palm forest that by the time of European contact had largely disappeared due to the impacts of land-clearing for agriculture and the invasive Pacific rat^66,67^. The duration and consequences of deforestation have been debated, with many arguing for an ecological catastrophe whereby rapid forest removal caused widespread erosion, depletion of soil productivity, reduction in surface freshwater, and reduced carrying capacity^29,60,68–70^. As part of these claims, paleoenvironmental evidence has been used to argue for a large-scale demographic change on pre-contact Rapa Nui, e.g.,^70,71^.

Recent paleoenvironmental studies also suggest that large-scale climate changes took place in pre-contact times. Sediment cores from Rano Raraku lake, for example, show a series of sedimentary hiatuses from the fifteenth to eighteenth centuries, leading some to argue that the island’s lakes became dry from severe droughts events^72,73^. These drought events are potentially associated with the onset of the Little Ice Age or changes in the El Niño Southern Oscillation, with a shift toward more positive Southern Oscillation Index (SOI) values beginning in the fifteenth century^74^.

Several studies have attempted large-scale analyses of chronometric dates to infer processes of cultural and demographic change on Rapa Nui. Using a set of ad hoc expectations for collapse, Mulrooney^75^ simulates SPD curves for population continuity and pre-contact collapse at ca. AD 1680 (the conventional collapse date). She compares these simulated results to an empirical SPD for settlement sites and concluded that the patterns did not meet expectations of a pre-contact collapse. Stevenson et al.’s^76^ SPD analyses of 428 obsidian hydration dates from six inland and coastal contexts similarly do not suggest a major population decline, from which they^76^ conclude that “this temporal reconstruction of land-use history associated with food production argues against the notion of an island-wide pre-contact collapse as a useful explanatory concept for Rapa Nui.” In an examination of the chronology for megalithic construction, DiNapoli et al.^57^ construct Bayesian tempo plots of available radiocarbon dates from statue platform (*ahu*) contexts. The results of these analyses demonstrate continuity in monument construction in pre-contact times, contrary to the common claims of collapse narratives^77^.

In a challenge to these analyses, Lima et al.^29^ present a series of SPD models they argue demonstrate demographic collapses prior to European arrival. Using normalized ^14^C dates and the *calsample* back-calibration method^23^, Lima et al.^29^ fit a linear growth model to the observed SPD and claim that deviations from the linear model show statistically significant “population collapses…at 1430–1550 CE…and 1640–1700 CE.” They also suggest that positive SOI values correspond to reduced local precipitation that stressed Rapa Nui’s rainfed cultivation systems and available surface freshwater. They then fit four logistic growth models directly to the normalized SPD, comparing simple logistic growth with three different models including effects from changes in palm cover estimated through palm pollen prevalence in lake cores, effects from changes in SOI that reflect overall dry/wet conditions, and a combination of palm cover and SOI. Lima et al. then compare these direct fits to the normalized SPD using IC (AIC and AIC_c_), from which they assert that the logistic model that includes strong effects from the reduction in palm cover and increasing SOI fits best. They argue that their finding demonstrates that climate variability and deforestation “describe the dynamics of the human population in Rapa Nui quite well and is able to explain the increasing trend as well as population decline episodes that impacted during several generations, which we think can be defined as demographic collapses.”

Several of Lima et al.’s^29^ modeling choices, however, raise questions about the validity of their conclusions. Their analysis hinges on the assumption that the rise and fall in normalized SPD at ca. AD 1500 represents an accurate demographic signal. The veracity of this assumption is unclear, however, as they did not examine the sensitivity of their results to ^14^C normalization or back-calibration simulation procedure. The shape of the Southern Hemisphere calibration curve from ca. 1375–1500 AD is steep, followed by a plateau and several ‘wiggles’^78,79^. Rather than reflecting demographic events, it is likely that this spike is a predictable consequence of normalization^23,46,47^. Critically, Lima et al.^29^ do not account for the calibration curve and sample size effects by fitting their models directly to the normalized SPD. As a result, their maximum likelihood calculations, the derived IC model selection, and conclusions are questionable. Instead, concluding that a collapse occurred may simply be explained as an artifact of their analytical procedure. Given these concerns, we are left with the question of how to account for paleodemographic patterns on Rapa Nui and how they may be related to past climatic and environmental changes. Here, we offer an ABC approach that reexamines these questions and is able to better account for the uncertainties that are inherent in SPD analyses.

## Results

**Fig. 2:**
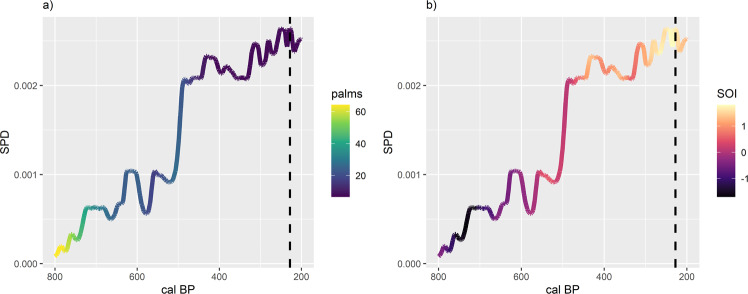
The observed non-normalized SPD calculated on the basis of 201 class 1 and 2 dates. The color of **a** shows changes in palm forest cover as measured by palm pollen abundance while the color of **b** shows changes in SOI. Positive SOI values are associated with drier conditions. The vertical dashed line marks the timing of European contact in AD 1722.

We reevaluate the hypothesis that Rapa Nui populations experienced an ecologically induced demographic collapse^29^. Figure 2 shows the non-normalized SPD curve generated from 201 radiocarbon samples from 47 archeological deposits on Rapa Nui (see “Methods”). Line color reflects changes in palm forest cover (Fig. 2a) and SOI (Fig. 2b). Based on the overall shape of the non-normalized SPD curve, one might conclude that populations experienced a logistic growth trend and that over time there was a corresponding decline in palm forest cover along with a shift from negative to positive SOI phases as the climate became drier. However, direct comparisons of the relationship between climatic variables and the SPD can yield opposite patterns by simply using normalized or non-normalized ^14^C dates (see Supplementary Note 2 and Fig. S1). This further affirms the danger of these direct comparisons and we must ask whether there is sufficient statistical basis to make such claims.

**Fig. 3:**
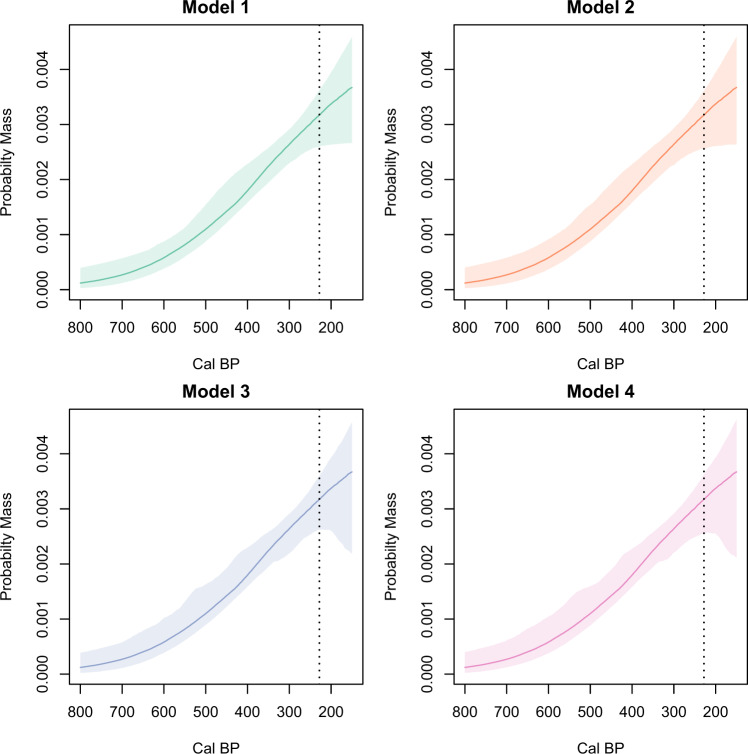
Median and 95% HPD values for four demographic models. Model 1 is a simple logistic growth curve, Model 2 is a logistic growth curve with a forest cover effect, Model 3 is a logistic growth curve with a climate effect from the SOI, and Model 4 is a logistic growth curve with palm forest cover and SOI. The colored areas indicate the 95% HPD envelope. The vertical dashed lines mark the timing of European arrival in AD 1722. Note that the HPD for all models includes the possibility of a demographic plateau after AD 1722, Models 3 and 4 include the possibility of a decline after European contact.

**Fig. 4:**
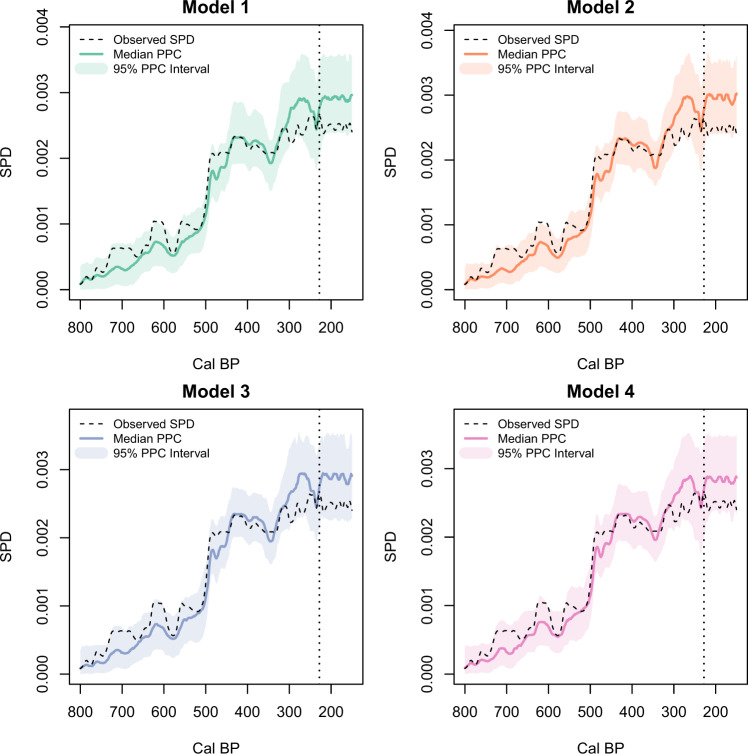
Posterior predictive checks (PPC) of the four fitted models against the observed Rapa Nui SPD. The dashed black line is the observed SPD. The solid color lines and shading show the median and 95% PPC interval for the fitted models. Note that the observed SPD lies within the simulation envelope of each model and the results of the four models are nearly identical. The vertical dashed lines mark the timing of European arrival in AD 1722.

To assess these human–environment interactions we used ABC to fit and compare four demographic models to the Rapa Nui SPD. These models include (1) simple logistic growth, (2) logistic growth with a palm forest cover effect, (3) logistic growth with a climate (SOI) effect, and (4) logistic growth with palm forest cover and SOI effects (see “Methods” and Supplementary Notes 1–11). Table 1 shows the median and 90% highest posterior density (HPD) estimates calculated for each model’s parameters based on the 1000 best-fitting runs. Checks of the joint posterior distributions show no correlations between model parameters (see Supplementary Note 8 and Figs. S10–S25). Figure 3 compares these fitted parameter values to demographic patterns. The four models show steady population growth from initial island settlement until European contact in AD 1722. Models 1 and 2 suggest that there was a potential population plateau following European arrival, whereas models 3 and 4 show possible decline after AD 1722. Posterior predictive checks of the fitted models against the observed Rapa Nui SPD are shown in Fig. 4 and indicate that the observed SPD lies within the simulation envelope for all four models.

**Table 1:** Median and 90% highest posterior density (HPD) estimates for models with non-normalized SPD using the *uncalsample* back-calibration method (see “Methods”). Using a normalized SPD and the *calsample* method produces comparable results (see SI).

	*N*_*t=0*_	*R*	*β*_pain_	*β*_SOI_
**Model 1** median (90% HPD)	0.06 (0.008–0.1)	0.008 (0.003–0.01)	–	–
**Model 2** median (90% HPD)	0.05 (0.006–0.1)	0.008 (0.003–0.01)	−0.001 (−0.017 to 0.015)	–
**Model 3** median (90% HPD)	0.06 (0.005–0.1)	0.009 (0.003–0.02)	–	0.074 (−0.28 to 0.4)
**Model 4** median (90% HPD)	0.06 (0.005–0.1)	0.009 (0.002–0.02)	−0.002 (−0.019 to 0.014)	0.082 (−0.28 to 0.4)

In contrast to Lima et al.’s^29^ AIC model comparisons showing strong support for model 4, our results show the four models are effectively identical and provide similar goodness-of-fit to the data, with Bayes factors not providing strong support for any particular model (Table S2). Posterior distributions of environmental parameters (*β*_palm_ and *β*_SOI_) for models 2–4 are also centered around zero with fairly broad 90% HPDs that range between positive and negative values. The substantial uncertainty in the posterior distribution and small Bayes factors are likely the consequence of the relatively small ^14^C sample for Rapa Nui that is not sufficient to discern between the competing hypotheses. These results provide strongly contrasting conclusions compared to those from direct comparison of the environmental proxies and the observed normalized or non-normalized SPDs. Failure to consider the factors included above gives conflicting and misleading results (see Supplementary Note 2).

## Discussion

When we assess the uncertainties of the Rapa Nui data and those involved in the analytic steps, the current evidence indicates that the island experienced relatively steady population growth from initial human settlement ca. 800 cal BP until the period following European arrival. The “wiggles” in the observed SPD curve all fall within the simulation envelope and result from details of the calibration curve combined with sampling error, and importantly, not genuine paleodemographic signals. Given these facts, we are unable to confidently distinguish between the four hypotheses. All of the fitted models, however, are consistent with a logistic growth pattern only marginally influenced by changes in climate and forest cover. The wide HPDs of the environmental parameters suggest a range of possible positive or negative effects, yet no values appear strong enough to cause major population declines (Fig. 3). Given the comparatively small number of radiocarbon dates, we cannot determine whether our inability to discern between the competing models is the consequence of small sample size, the small ‘effect size’ in models 2–4 (i.e., the absolute deviation of *β*_palm and_ *β*_SOI_ from 0), or a combination of both factors. Nonetheless, none of the fitted models support the notion of pre-contact population collapse (Fig. 3). Therefore, our results suggest that if deforestation or increasing SOI had effects on the island, Rapa Nui populations were resilient to them. These findings are independently supported by recent research showing that monument construction steadily continued even after European arrival^57,77^. In addition, research now demonstrates that deforestation was a prolonged process, did not result in catastrophic erosion, and that land cover was quickly replaced by lithic mulch gardens that increased agricultural productivity^66,67,80–85^. Moreover, while some claim that deforestation resulted in the loss of food^29,68^, there is no evidence that palms were a significant dietary resource for islanders^66,86^. Thus, it is more likely that the loss of the palm forest represented an expansion of cultivation opportunities and positively contributed to the initial growth and overall resilience of the population. In summary, there is no empirical support for the notion that deforestation resulted in strong negative impacts on the human population of Rapa Nui.

Our results also have implications for the effects of climate change on the island. Rull^71,73^ has recently claimed that climate-induced droughts caused a large-scale societal disruption resulting in the cessation of monument construction and intra-island migration from coastal settlements to the crater lake at Rano Kau. Similar to previous analyses of the tempo of monument construction around the island^57^, the vast majority of our ^14^C data derive from coastal settlements and do not show declines in activity or support claims of major climate-induced disruptions from drought. While climate perturbations seem to have led to desiccation of the crater lake at Rano Raraku^72^, recent research suggests Rapa Nui populations adapted to these changes by relying primarily on coastal groundwater sources^87–89^.

Our analyses also provide important insights into previous demographic proposals by Puleston et al.^64^. In a series of hypothetical food-limited demography simulations, Puleston et al.^64^ derived several absolute estimates for maximum population sizes on pre-contact Rapa Nui, which converged on two possible outcomes largely dependent on assumptions of bioavailable nitrogen for cultivation: a ‘low-N scenario’ with maximum populations of ca. 3500 and a ‘high N scenario’ with maximum populations of ca. 17,500. Because at the time of initial contact, Europeans estimated the population to be in the low thousands^63^, Puleston et al.’s^64^ conclusion of 17,500, if correct, logically requires a large pre-contact demographic decline^65^. Our study provides a resolution to this debate as none of the models show evidence of a major reduction in activity prior to European arrival, indicating that Puleston et al.’s^64^ low-N estimate of ca. 3500 individuals is most consistent with the archeological and historical evidence. At a broader scale, our results demonstrate that the logistic demographic patterns on Rapa Nui were similar to those proposed for other islands in Polynesia, e.g.,^59,90–92^.

Combined with a growing body of recent research, our demographic analyses support an emerging view of Rapa Nui as a case of population resilience in the face of marked environmental changes, e.g.,^58,61–63,75–77,93–95^. These results present a history that stands in contrast to the popular narrative of sequential catastrophic events, which has inflated environmental changes as negative drivers of cultural and demographic patterns. Rather, despite extreme isolation, marginal ecological conditions, and a series of environmental changes, Rapa Nui people found solutions that enabled them to successfully thrive on the island for at least 500 years prior to the arrival of Europeans.

In summary, our conclusions sharply contrast with Lima et al.’s^29^ study and falsify claims of a population decline prior to European contact. Several factors explain the divergence between the conclusions of Lima et al.’s^29^ study and those presented here, along with those of previous studies^57,75^. First, Lima et al.’s study did not correctly ‘bin’ dates by site location. While samples in Mulrooney’s^75^ dataset are coded by ‘site,’ archeological site-naming conventions yield instances where dates from the same site have slightly different site names. For example, the large sample of dates from the Anakena settlement is represented by ~15 ‘sites’ in Lima et al.’s study, when in fact all dates come from excavations within or closely adjacent to the site of Ahu Nau Nau. Second, while Lima et al.^29^ correctly note that samples from ceremonial contexts, e.g.,^57^ reflect “the ‘ahu moai’ tradition” and “not a demographic process”, their study nevertheless included ca. 70 ^14^C dates from these contexts that by their own admission are likely unrelated to population changes. These two issues result in an ambiguous relation between dates and target events while also inflating the sample size.

Importantly, Lima et al.’s study incorrectly describes the results of the linear model that provides a foundation for their study’s collapse hypothesis^29^. Through their choice of a linear model, steps by which they normalized the ^14^C data, and confusion over the effects of the calibration curve, Lima et al.’s^29^ study misinterprets variability in the SPD. The rise and fall (i.e., “collapse”) observed in their study between 1430 and 1550 AD falls entirely within a significant positive deviation from their null model. Given the known impact of ^14^C normalization at this steep portion of the Southern Hemisphere calibration curve^23,46,47,75,78,79^, the population only appears to rise and decline. Moreover, Lima et al.’s study does not provide a clear rationale why continuous linear population growth would be expected for the small island of Rapa Nui. Perhaps most critically, by not adequately accounting for calibration or sample size effects in their attempt to directly fit their regression models to the normalized SPD, Lima et al.’s study^29^ treated these artifacts of the calibration curve as genuine demographic signals. An important lesson of Lima et al.’s^29^ study for future work is that by not acknowledging different forms of uncertainties, avoiding direct assessment of SPDs is not just a matter of statistical rigor, but an issue that can lead to dramatically different claims about the past.

From a methodological standpoint, the generative inference approach provided by ABC offers a solution that addresses the need to evaluate complex demographic models with sufficient statistical rigor while also taking into account the specific challenges of radiocarbon datasets. The main advantage of the ABC approach for model fitting and comparison over recently suggested approaches^33,34,45^ is the flexibility offered by formulating the proposed model as a simulation, an advance that could potentially integrate more complex phenomena involved in shaping SPDs such as settlement dynamics^96^ and geoarchaeological concerns such as taphonomy^97^ and variable sedimentation rates^25^. While the price for this flexibility is the relatively high computational requirements, this factor is outweighed by the benefits gained from more fully characterizing analytical uncertainty, opening up opportunities for empirically testing an increasing number of computational models, and ultimately improving the accuracy of the inferential process in archeology.

## Methods

### Radiocarbon and paleoenvironmental data

We use the ^14^C dataset compiled in Mulrooney^75^ along with recently published dates^93,98,99^, including all radiocarbon determinations meeting the criteria for class 1 or 2 dates by Mulrooney^75^. Following Brown and Crema^90^, we restrict our analysis to dates with a clear contextual association with residential sites or subsistence activity to ensure linkage between the dated events and our target phenomena of past demography. Samples come from across the island and derive from similar sedimentary contexts resulting from anthropogenic depositional events^75^, cf. ^25^. These steps resulted in 201 dates from 47 locations. We calibrated all terrestrial dates with the SHCal20 calibration curve^79^. All bone dates were calibrated with a mixed terrestrial/marine curve with 50% SHcal20 and Marine20^100^ following Jarman et al.^93^ and a revised local marine reservoir correction. There are currently ten local marine reservoir corrections (Δ*R*) for the island, all of which derive from coral samples from Ovahe, a small bay just east of Anakena^101,102^. These Δ*R* values range from −132 ± 37 to −252 ± 46. We revised the Δ*R* values to reflect updates to the Marine20 calibration curve^100^. Following methods in DiNapoli et al.^103^, we calculated the error-weighted pooled mean with external variance added for these Δ*R* values to be −214 ± 16 (*χ*^2^_9:0.05_ = 11.0 < 16.9; $\frac{{{{\chi }}}^{2}}{n-1}\,$= 1.2).

To examine the effects of ^14^C normalization on demographic interpretations, we generated two SPD curves for the time period 800–150 BP with both normalized and non-normalized dates. To account for ascertainment and wealth bias^34^, dates from the same site and within a temporal distance of 50 years were combined into bins. To examine possible taphonomic biases we applied the correction suggested by Surovell et al.^104^ to the normalized and non-normalized SPD, which does not result in an observable difference in the SPDs (Fig. S2). We interpolated time-series data on changes in forest cover using palm pollen counts from paleoenvironmental cores of Rano Raraku lake sediments^72^, and SOI reconstructions from Yan et al.^74^, the same datasets underlying Lima et al.’s^29^ analyses.

### Demographic models

Given Rapa Nui’s small size, we assume that population growth on the island followed some form of logistic pattern. Following Lima et al.^29^, we constructed four demographic models with the following general structure:

1 $${N}_{t}={N}_{t-1}\ast {e}^{r\ast [1-({N}_{t-1}/{K}_{t-1})]}$$

where $N$ is the population size, $t$ is time, and $r$ is intrinsic growth rate, and $K$ is carrying capacity. The four models are distinguished by the parameters and covariates of the linear model defining ${K}_{t-1}$ :

2 $${K}_{t-1}={\rm{exp}}({\beta }_{{\rm{palm}}}{F}_{t-1}+{\beta }_{{\rm{SOI}}}{C}_{t-1})$$

where $F$ is forest cover, $C$ is SOI climate reconstruction, and *β*_palm_ and *β*_SOI_ are model parameters. Model 1 has all parameters equal to zero, and corresponds to a simple logistic growth model with a carrying capacity equal to 1. The second and third logistic models incorporate either palm cover (by setting *β*_SOI_ = 0) or the SOI index (by setting *β*_palm_ = 0) individually, whilst the fourth model explores the impact of both external covariates.

### Approximate Bayesian Computation for SPD modeling

Our ABC approach to SPD modeling builds on recent work by Porčić^56^, follows standard procedures used in other fields, e.g.,^105,106^, and involves the following steps: (1) define the prior distributions of the demographic model parameters; (2) sample *n* parameter combinations from the prior distributions; (3) define the target SPD via random thinning; (4) simulate SPDs from each combination; (5) compare the goodness-of-fit between each simulated and observed SPDs using some distance measure ε (e.g., Euclidean distance); (6) reject parameterizations that deviate from a user-defined cutoff value for the distance measure. The result of these steps is an approximated joint posterior distribution of parameter values most consistent with the observed radiocarbon dataset. We then compare the fit of models using approximate Bayes factors, computed from the relative proportion of the four models in the non-rejected ensemble.

The core assumption of the so-called dates as data approach^107^ can be formalized as follows:

3 $${p}_{t}\propto {N}_{t}$$

where ${p}_{t}$ is the probability of sampling a radiocarbon date from the calendar time $t$. For a given model and parameter values, this is achieved by calculating the vector of population sizes ${N}_{t}$ and dividing each of its values by the sum of all population sizes within the window of analysis, and setting the initial population size as the parameter ${N}_{t}=0$, which can be interpreted as the proportion of the carrying capacity for the baseline model (i.e., when *β*_SOI_ = *β*_palm_ = 0). Thus, our core generative framework consists of sampling radiocarbon dates using the vector ${p}_{t}$. Following Crema and Bevan^23^, we use two different algorithms (*calsample* and *uncalsample*) for transforming the vector ${p}_{t}$ into a vector of probabilities in ^14^C age. While in other contexts the two approaches yields different results (with *uncalsample* recovering more accurately the artificial spikes typical of some observed SPDs), our results do not provide any qualitative differences (see Supplementary Notes 4 and 7–11).

The key steps of our ABC approach are summarized as follows:

Step 1: The prior distributions for each of our demographic models are shown in Table S1. The initial population size, *N*_*t=0*_, is truncated to an upper bound of 1 to specify that we assume the population size of the island at 800 cal BP to be below carrying capacity. We assume a positive intrinsic growth rate with a conservative magnitude that ensures that half of the draws are below a rate of ca. 0.01. Given ongoing debates about the potential impacts of changes in forest cover and SOI on the island’s carrying capacity, the prior distributions for the palms coefficient, *β*_palm_, and the SOI coefficient *β*_SOI_ can take a range of positive and negative values. The benefit of these relatively flat priors is that the ABC rejection algorithm will select the parameter values that are most consistent with the empirical SPD patterns. The prior parameter combinations for each model are visually assessed via prior predictive checks (Fig. S4).

Step 2: We sample 250,000 parameter combinations from the prior distributions of each of our four models.

Step 3: We sample a single radiocarbon date from each temporal bin and generate a target SPD via summation and compute the proportion of dates that were calibrated using SHCal20 and the mixed SHCal20/Marine20 calibration curve.

Step 4: We generate a vector of population sizes ${N}_{t,i}$ for each *i*th parameter combination ${\theta }_{i}=\{{N}_{t=0,i},{r}_{i},{\beta }_{{\rm{palm}},i},{\beta }_{{\rm{SOI}},i}\}$ using Eqs. 1 and 2, and obtained a vector of probabilities ${p}_{t,i}$ following the procedure described above (see Fig. S3 for a diagram of this process). We then sample 110 radiocarbon dates (corresponding to the number of *bins*) that are each randomly assigned an error term by resampling from the observed ^14^C errors, calibrate each date, and generate a candidate SPD_*i*_. To evaluate the sensitivity to various algorithms and assumptions in the literature, we sample radiocarbon dates using the *calsample* and *uncalsample* algorithms, and calibrate dates with and without normalization. *Calsample* refers to the procedure where dates are simulated from the null model in calendar time and then back-calibrated, whereas in the *uncalsample* method the entire null model is back-calibrated, weighted based on a uniform model, and then sampled from^23^. Thus, for each parameter combination, we generate four SPDs depending on the combination of these procedures. We have not identified any qualitative differences between these different combinations of algorithms for the present study (see Supplementary Notes 4 and 7–11). Both the sampling procedure and the calibration process were carried out using SHCal20 and the mixed SHCal20/Marine20 curves based on the proportions obtained during Step 3.

Step 5: We then compare the fit between each simulated SPD_*i*_ and the observed SPD using two error measures *ε*, Euclidean distance and normalized root-mean-square error (see Supplementary Note 6). To ensure comparability between candidate and observed SPDs, we generate the latter at each *i*th iteration by randomly sampling a calendar date within each *bin* before summation.

Step 6: We obtain posterior distributions of our model parameters by selecting the combinations yielding the 1000 lowest *ε* values for each model. Similarly, we compare the relative fit of all models by combining all the ε values and selecting the 1000 lowest *ε* values across the four models. The relative proportions of models 1–4 in this set of 1000 best-fitting models are then used to compute approximate Bayes factors, which provide an estimate of the relative weight of evidence in favor of one model over another.

Step 6: We further evaluate the absolute goodness-of-fit between our four models and the observed SPDs by posterior predictive checks. This step consists of visual comparison of the observed SPDs and an envelope generated from the 95th percentile interval of predicted SPD values for each calendar year based on the 100 best-fitting parameter combinations. All of our analyses are conducted in R version 4.0.3^108^ using the *rcarbon* package^23^. Fully reproducible code, along with a Shiny app to explore the models’ dynamics, can be found at https://github.com/rdinapoli/RN_demography

### Reporting summary

Further information on research design is available in the Nature Research Reporting Summary linked to this article.

## Supplementary information

Supplementary Information

Reporting Summary

## Data Availability

All data necessary to reproduce these analyses are available at https://github.com/rdinapoli/RN_demography and 10.5281/zenodo.4883617

## Code availability

All code necessary to reproduce these analyses are available at https://github.com/rdinapoli/RN_demography and 10.5281/zenodo.4883617
