# Supplementary material for: Approximate Bayesian Computation of radiocarbon and paleoenvironmental record shows population resilience on Rapa Nui (Easter Island)
Source: Nat Commun. 2021 Jun 24;12:3939. doi: 10.1038/s41467-021-24252-z (PMC8225912; doi:10.1038/s41467-021-24252-z)
Supplement: Supplementary file 1 — Supplementary Information [file 41467_2021_24252_MOESM1_ESM.pdf]

Supplementary Information for:

Approximate Bayesian Computation of radiocarbon and paleoenvironmental record shows population resilience on Rapa Nui (Easter Island)

Robert J. DiNapoli[1,*], Enrico R. Crema[2], Carl P. Lipo[1], Timothy M. Rieth[3], Terry L. Hunt[4]

1. Environmental Studies Program, Department of Anthropology, Harpur College of Arts and Sciences, Binghamton University, State University of New York

2. Department of Archaeology, University of Cambridge

3. International Archaeological Research Institute Inc., Honolulu

4. The Honors College and School of Anthropology, University of Arizona

*Corresponding author: Robert J. DiNapoli, Email: dinapoli@binghamton.edu

Table of Contents

Supplementary Note 1: Introduction 1

Supplementary Note 2: Normalization. 2

Supplementary Note 3: Taphonomic bias 3

Supplementary Note 4: Back-calibration methods. 3

Supplementary Note 5: Prior predictive checks 4

Supplementary Note 6: Distance measures for ABC rejection algorithm. 7

Supplementary Note 7: Posterior predictive checks 8

Supplementary Note 8: Joint posterior distributions 11

Supplementary Note 9: Marginal Posterior Distributions 27

Supplementary Note 10: SPD Posterior Predictive Checks 37

Supplementary Note 11: Bayes Factor Matrices 42

Supplementary References 44

Supplementary Note 1: Introduction

Here, we provide a series of supplementary analyses that explore the sensitivity of our results to various modeling choices. We explore the effects of three primary analytical decisions: (1) the impact of normalized versus non-normalized calibrated radiocarbon dates; (2) the choice of back-calibration simulation procedure used to fit the demographic models to the observed SPD; and (3) the distance measure used in the Approximate Bayesian Computation (ABC) rejection algorithm, specifically Euclidean distance and normalized root mean squared error. As demonstrated in the supplementary results below, our conclusions are robust to these different modeling choices.

We conducted all of our analyses in R version 4.0.3 [1] using the *rcarbon* 1.4.1 package [2]. All data and fully reproducible code can be found at https://github.com/rdinapoli/RN_demography. Note that the ABC model fitting procedure is computationally intensive and requires hours to days to complete depending on computational resources.

Supplementary Note 2: Normalization.

As discussed in the main text, normalizing radiocarbon dates during calibration can cause spurious spikes in SPDs at steep portions of the calibration curve [2–4]. We thus explored the fit of the demographic models using both normalized and non-normalized radiocarbon dates.

To illustrate the dangers of directly comparing SPDs to environmental proxies, and the divergent results one might obtain by normalizing ^{14}C dates, Figure S1 compares the apparent relationship between the observed normalized and non-normalized SPDs with forest cover and SOI. If we partition the SPD into periods prior to and following Lima et al.'s [5] proposed collapse event, which also coincides with major reductions in forest cover and shifts to positive SOI phases, the normalized and non-normalized SPDs yield contradictory patterns. Prior to ca. 500 cal BP the normalized SPD shows a strong positive correlation with palm cover (*rho* = 0.8) and SOI (*rho* = 0.7), whereas after this the relationship with palm cover weakens (*rho* = 0.3) and reverses for SOI (*rho* = -0.5). In contrast, the non-normalized SPD shows a consistent negative relationship with palm cover and a positive relationship with SOI. The normalized SPD thus suggests that relative population declined with decreasing forest cover and increasing SOI, whereas the non-normalized SPD suggests precisely the opposite relationship. Because such direct comparisons do not account for sampling and measurement errors, null hypothesis tests can lead to overconfidence in misleading results, and in this case, to inflated and infinitesimal P-values.

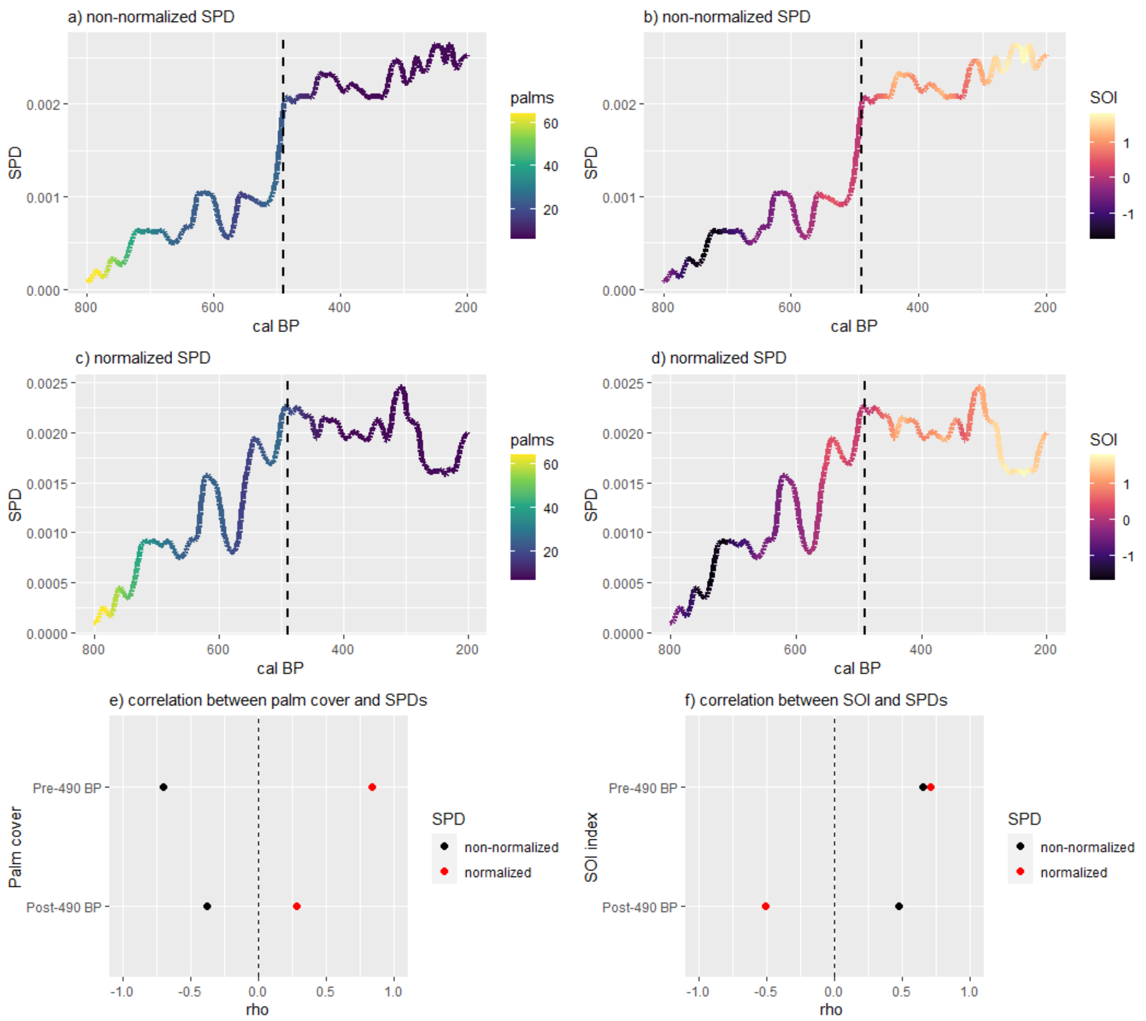

Figure S1.The relationship between the observed Rapa Nui SPDs and changes in forest cover and SOI index. a) non-normalized SPD as a function of palm forest change, b) non-normalized SPD as a function of changing SOI index, c) normalized SPD as a function of palm forest change, d) normalized SPD as a function of changing SOI

index. The black vertical dashed line is the approximate timing of Lima et al.'s [5] proposed collapse event that coincides with major changes in SOI and forest cover. e) showing the correlation between palm cover and the normalized and non-normalized SPD before and after this proposed collapse, and f) shows the correlation with SOI index.

Supplementary Note 3: Taphonomic bias

To explore the potential impact of taphonomy on the shape of the observed SPDs, we applied the correction of Surovell et al.[6] to both the normalized and non-normalized SPD. Figure S2 shows the results and suggests no meaningful observable difference between the original and taphonomically corrected SPD.

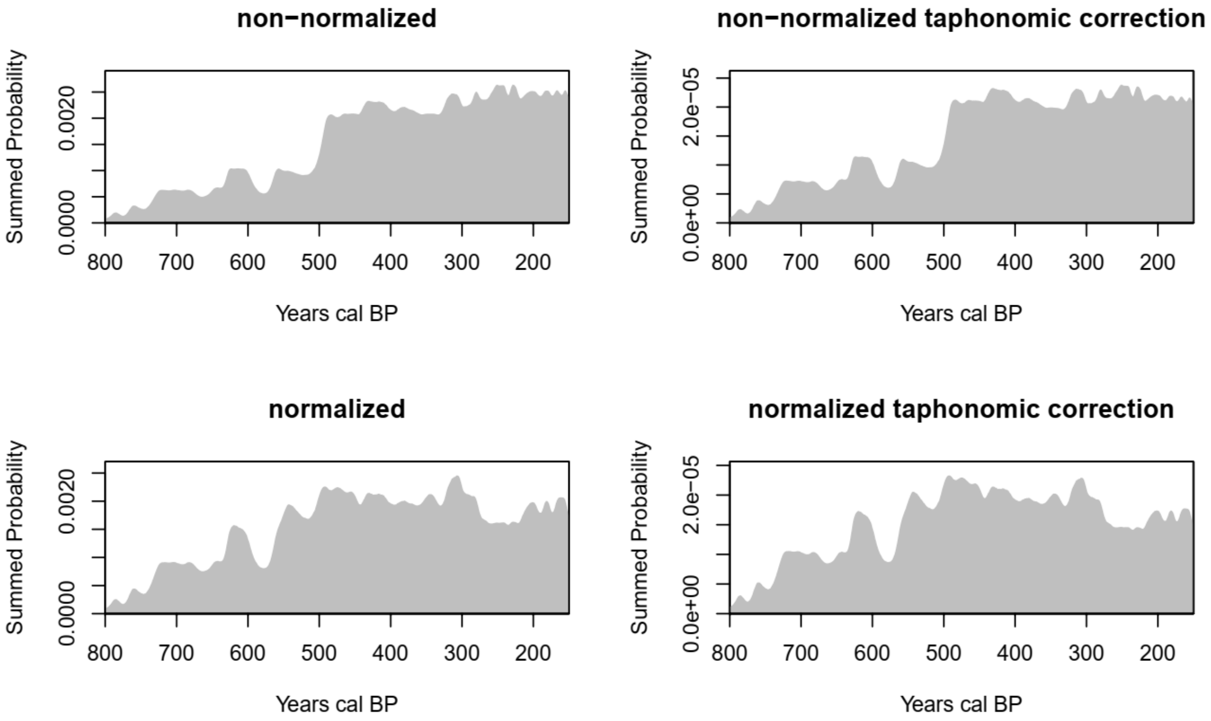

Figure S2.Comparison of taphonomically adjusted and non-adjusted SPDs for the Rapa Nui dataset.

Supplementary Note 4: Back-calibration methods.

A further complication of SPD model fitting is the ability to simulate theoretical SPDs of radiocarbon dates consistent with the proposed model and capable of emulating features characteristic of empirical SPD dictated by calibration effects. Commonly, this procedure consists of randomly sampling dates from a vector of probability in calendar time followed by back-calibration, and calibration. Crema and Bevan [2] have shown that for discrete time-steps this result can be achieved by calibrating the entire vector of probability (see equation 1 in Crema and Bevan [2]) and sampling directly in ^{14}C age. However, this approach, referred to as *calsample*, does not consistently recover artificial peaks typically observed in SPDs. Bevan et al.[7] have devised an alternative algorithm (*uncalsample*) that applies a weighting scheme based on a uniform probability model. In order to explore the sensitivity to these alternative algorithms, we used both *calsample* and *uncalsample* approaches in our calculations.

Supplementary Note 5: Prior predictive checks

Table S1 shows the prior distributions of the demographic model parameters as described in the Methods section of the main text.

Table S1. Prior distributions of the demographic model parameters.

**Symbol**	**Description**	**Prior**	**Relevant model**
$N_{t=0}$	Initial population as a proportion of carrying capacity	Exponential ~ (λ=10)	All
r	Intrinsic growth rate	Exponential ~ (λ=50)	All
β_{palm}	Scaling parameter on palms	Gaussian ~ (0, 0.01)	2,4
β_{soi}	Scaling parameter on SOI	Gaussian ~ (0, 0.2)	3,4

Figure S3 shows a diagram of the demographic model structures. For each simulation from a model, a prior parameter value is selected and used to generate a vector of population sizes (N_t) for the time period of interest, which is transformed into a probability density function that is compared to the observed SPD. The four bottom panels in Figure S3 show examples of different possible model outcomes depending on the particular prior values.

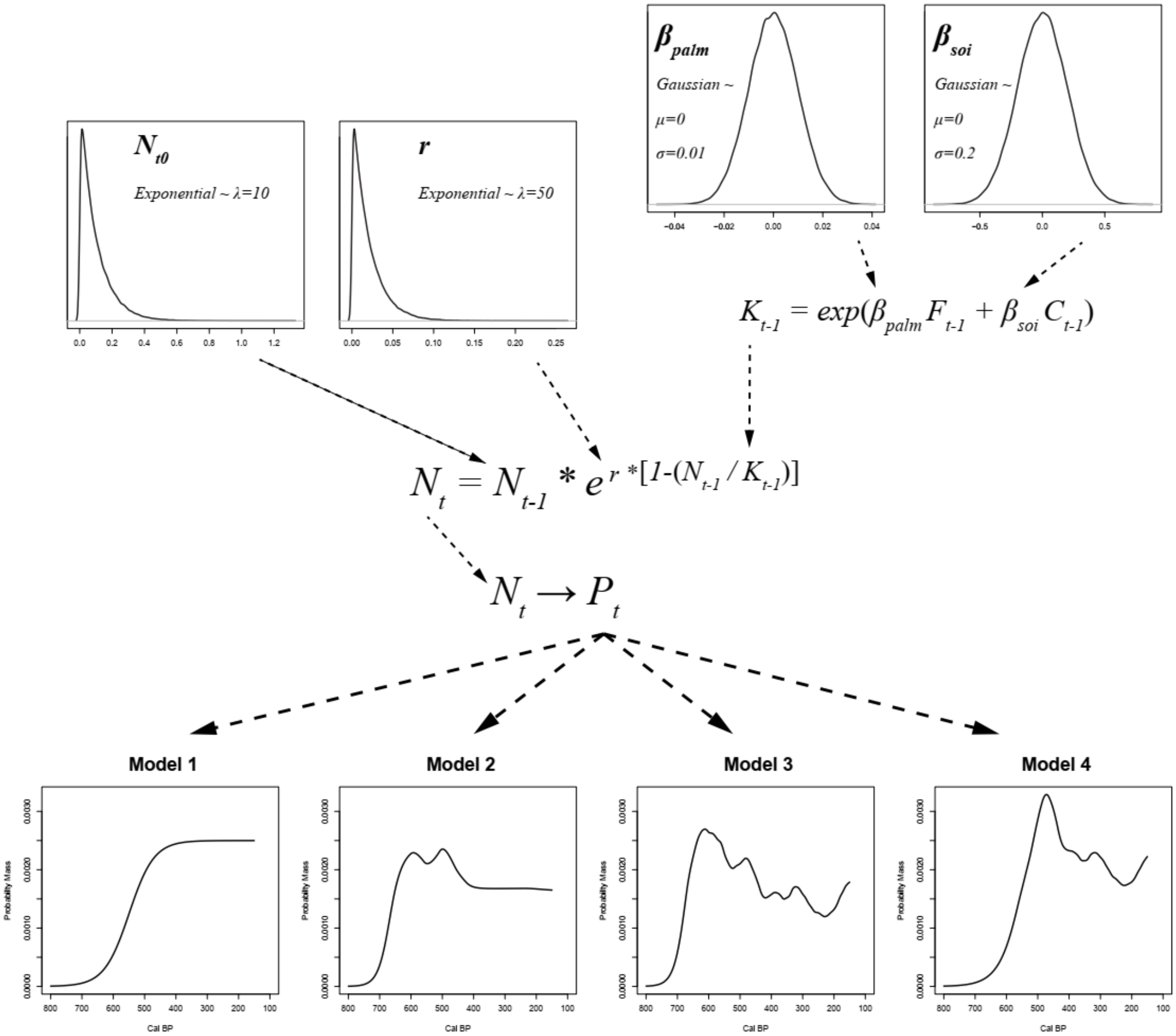

Figure S3. Diagram of demographic model structure. The prior values used for this illustration are as follows: Model 1 (N_{t0}=0.005, *r*=0.025), Model 2 (N_{t0}=0.005, *r*=0.05, β_{palm}=0.02, β_{soi}=0), Model 3 (N_{t0}=0.005, *r*=0.05, β_{palm}=0, β_{soi}=-0.4), Model 4 (N_{t0}=0.005, *r*=0.025, β_{palm}=0.02, β_{soi}=-0.4).

The plots below show prior predictive checks based on 1,000 simulated iterations of models 1-4.

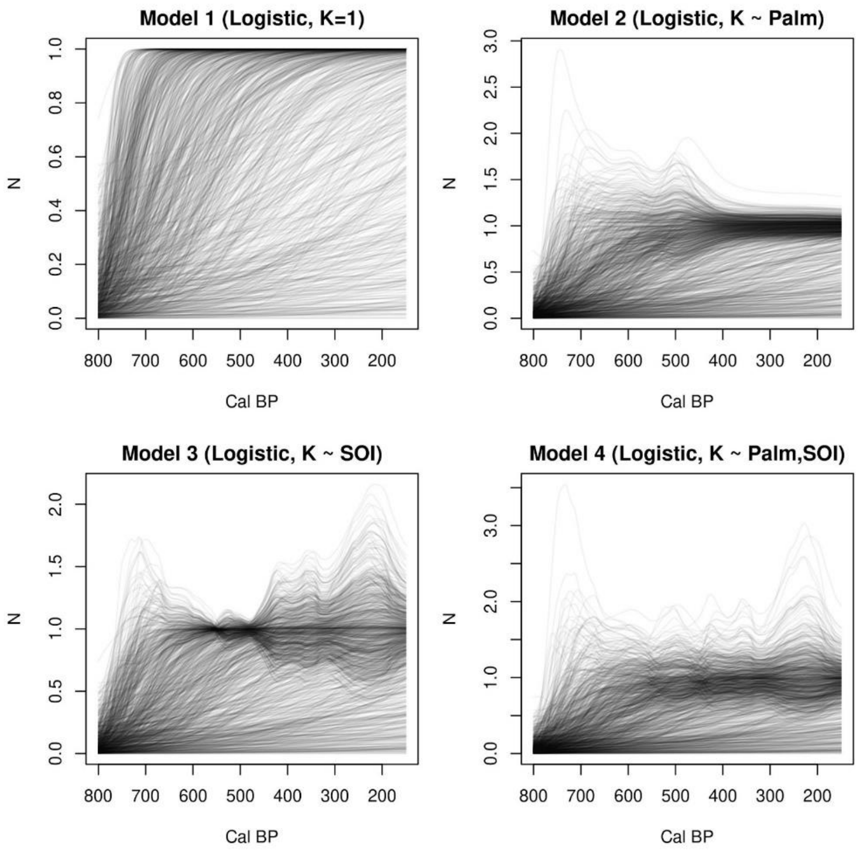

Figure S4. Prior predictive checks for models 1-4 by population size (N_t).

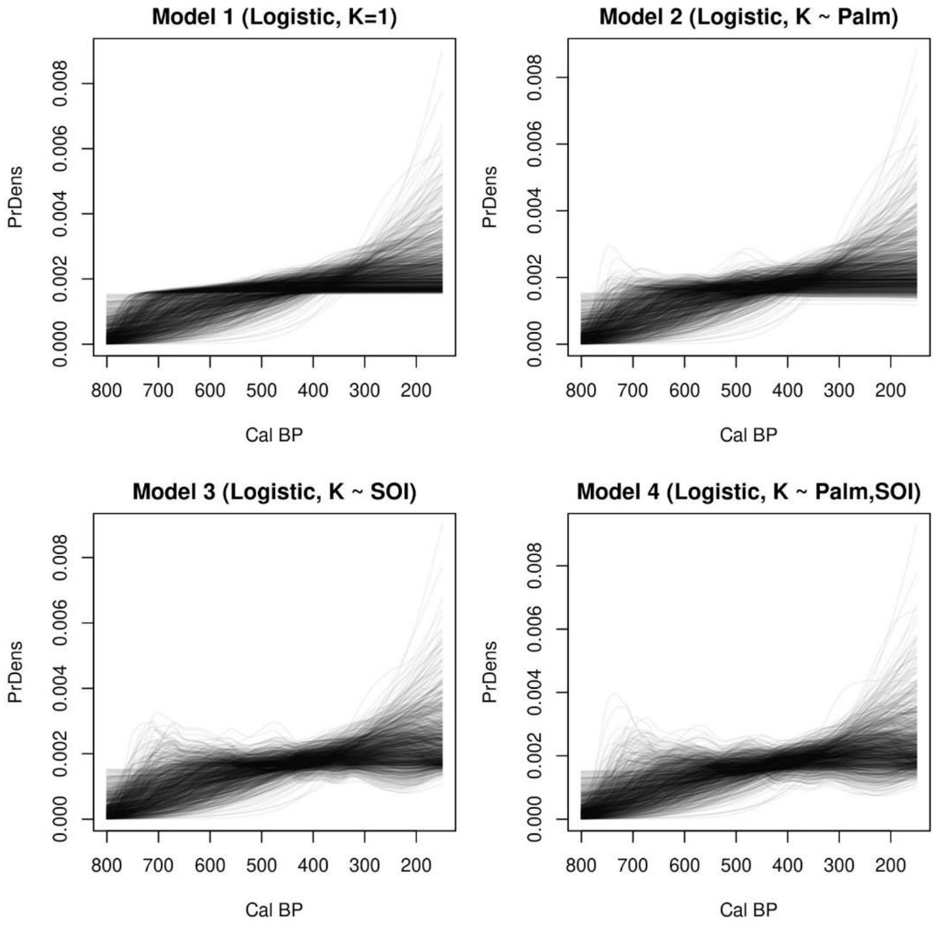

Figure S5. Prior predictive checks for models 1-4 by probability density.

Supplementary Note 6: Distance measures for ABC rejection algorithm.

The rejection algorithm that underlies the ABC model fitting procedure requires a measure of distance between each realization of the models and the observed SPD. As discussed in previous studies [2] the observed and simulated SPDs can be treated as test statistics that can be directly compared. Here we use two measures of distance— Euclidean distance and normalized root mean squared error (NRMSE). Euclidean distance (ε_E) is calculated with the following formula:

$$\varepsilon_E = \sqrt{\sum_{t=1}^{T}(S_{o,t} - S_{s,t})^2} \quad (1)$$

where $S_{o,t}$ is the probability density of observed SPD at time t and $S_{s,t}$ is the probability density of a simulated SPD from the model at the same timestep.

NRMSE (ε_N) is calculated with:

$$\varepsilon_N = \frac{\sqrt{\sum_{t=0}^{T}(S_{o,t} - S_{s,t})^2}}{T/\sigma S_o} \quad (2)$$

where T is the length of time in the model and σS_o is the standard deviation for the probability density of the observed SPD. For both distance measures, smaller values indicate a closer fit between the realization of the model and the observed data.

Supplementary Note 7: Posterior predictive checks

Below, we present the posterior predictive checks for each iteration of models 1-4. All results below are based on a rejection algorithm using Euclidean distance. Using normalized root-mean-square-error produces nearly identical results, which can be found at https://github.com/rdinapoli/RN_demography.

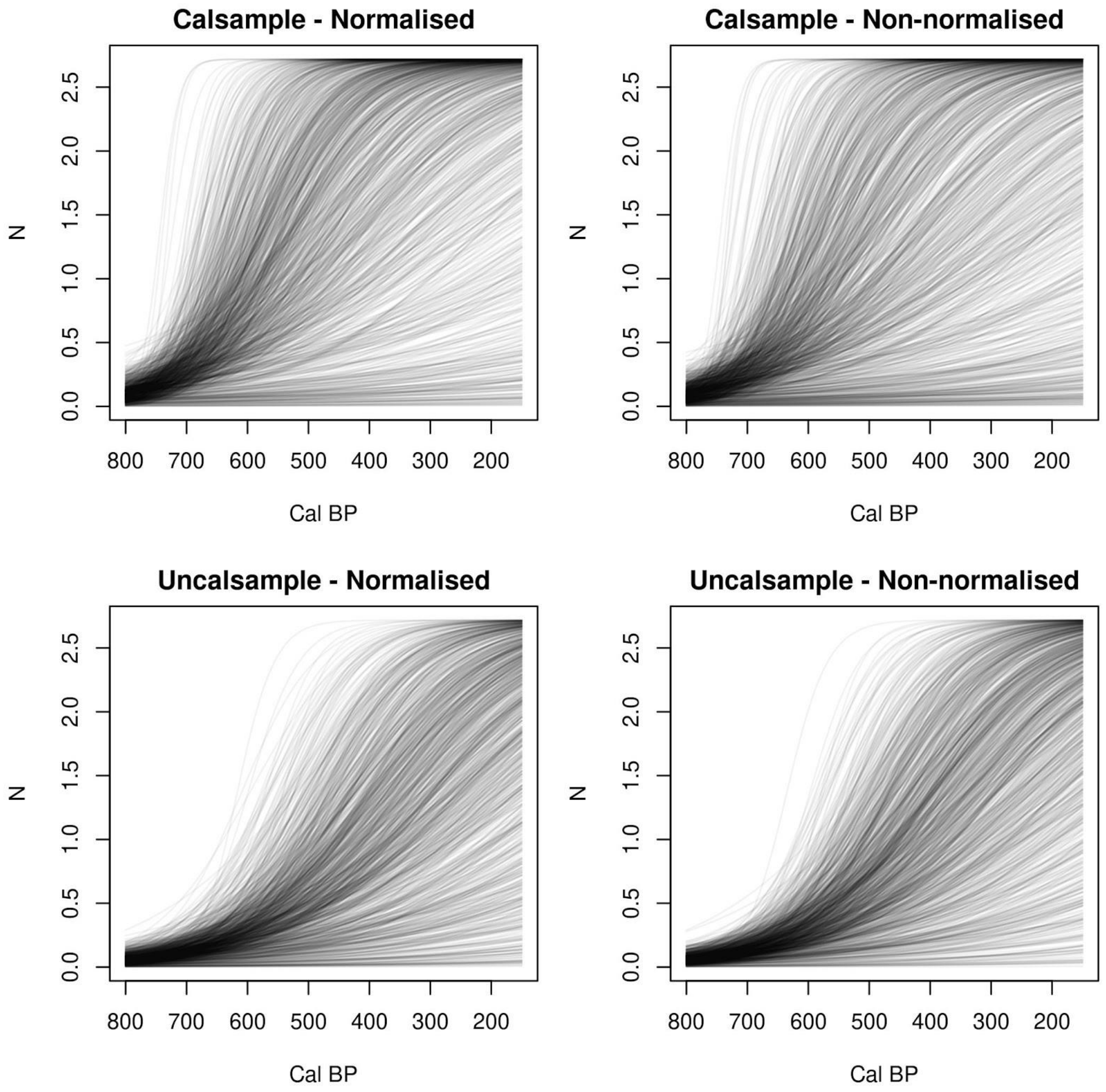

Figure S6. Posterior predictive check for model 1 using Euclidean distance.

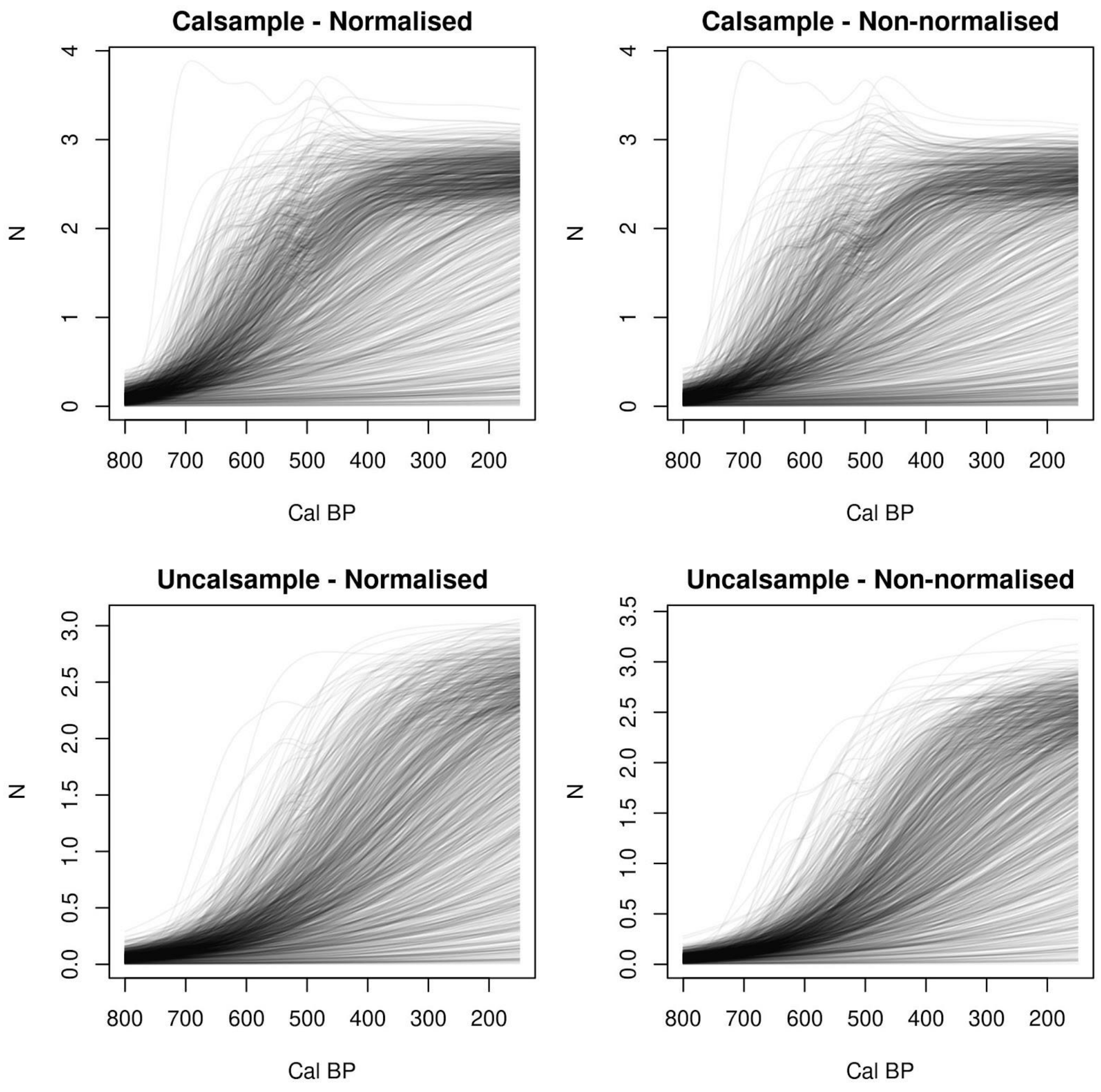

Figure S7. Posterior predictive check for model 2 using euclidean distance.

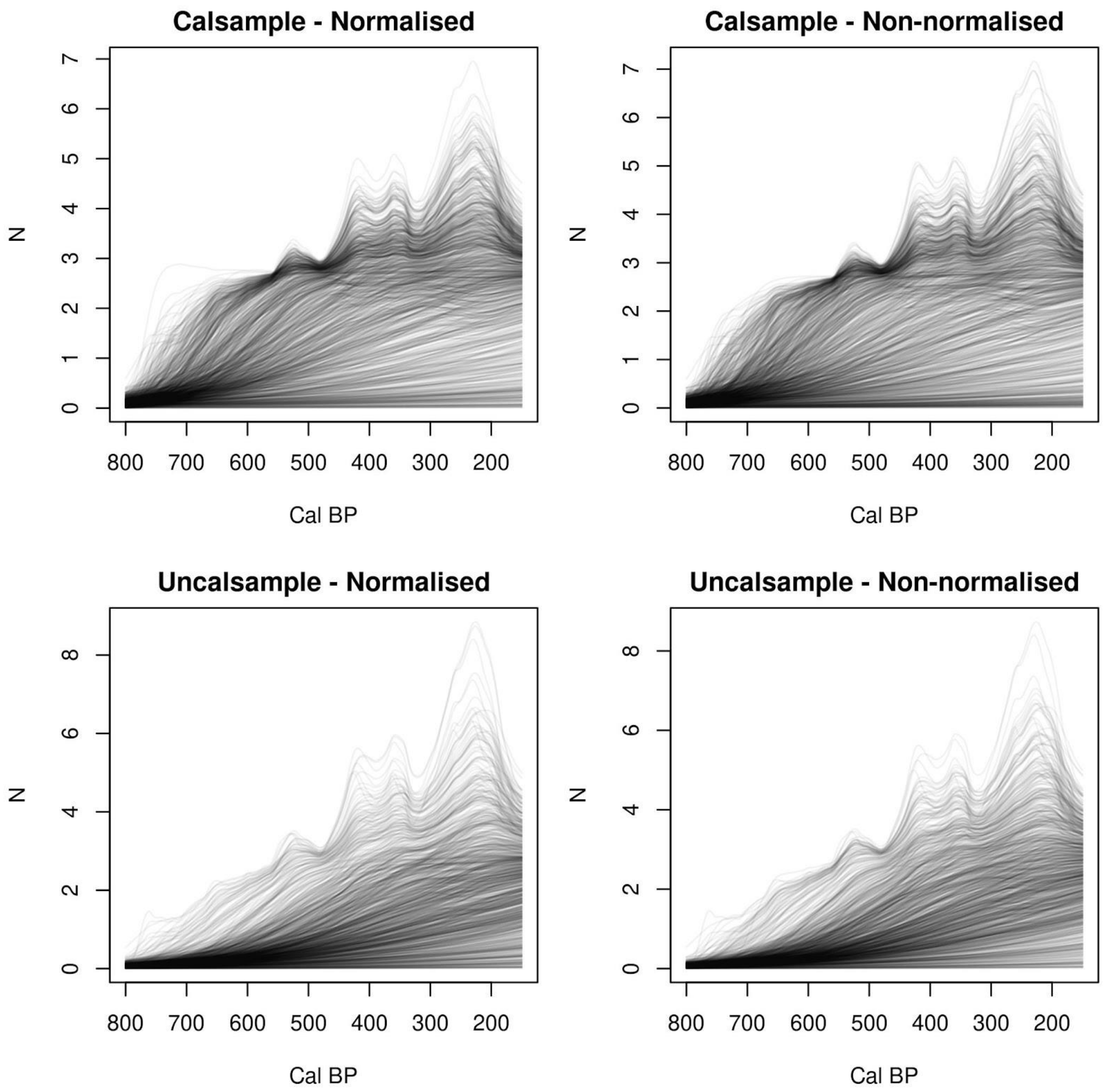

Figure S8. Posterior predictive check for model 3 using euclidean distance.

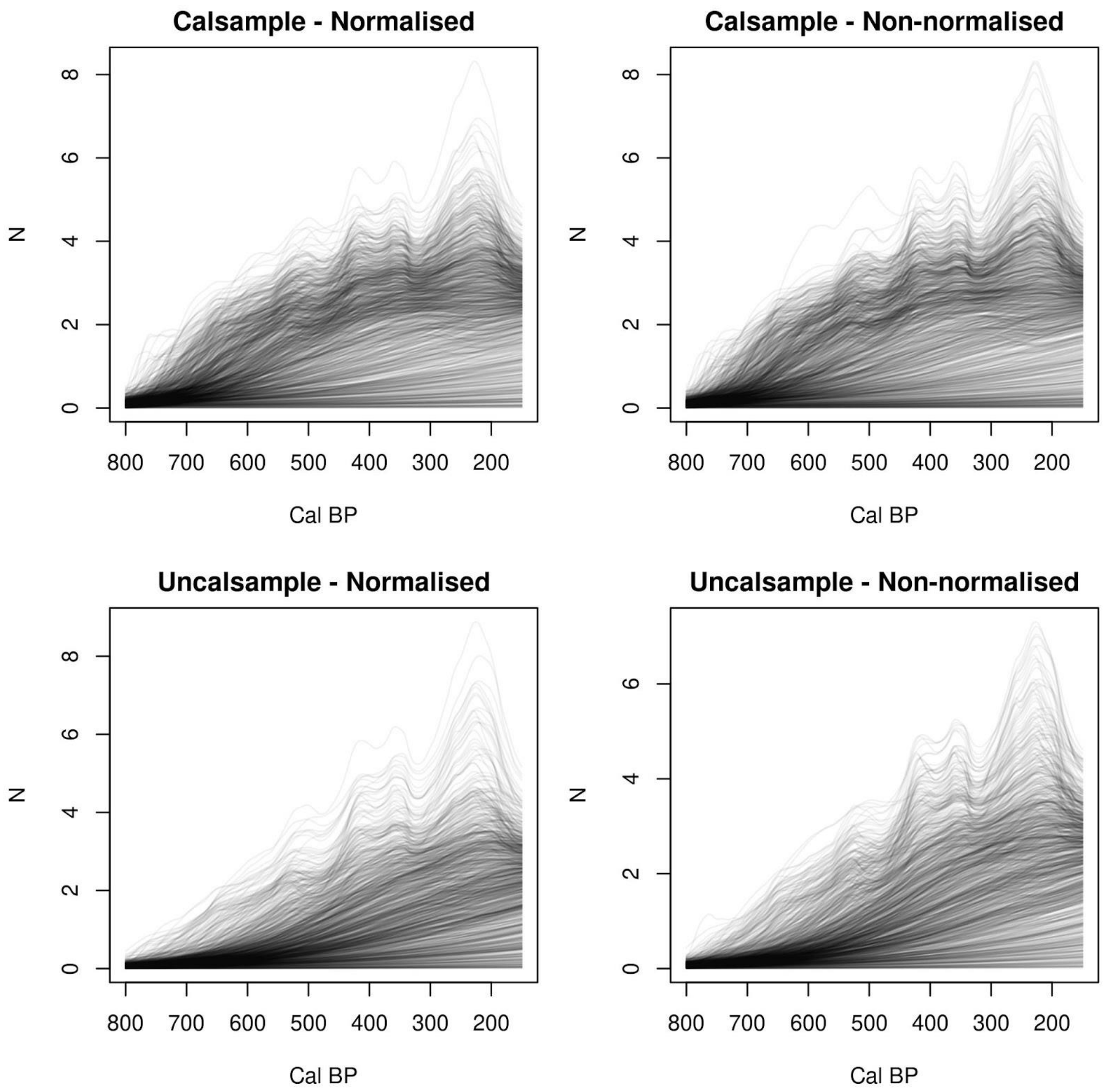

Figure S9. Posterior predictive check for model 4 using euclidean distance.

Supplementary Note 8: Joint posterior distributions

Below, we present the joint posterior distributions of the parameters for each model to check for potential collinearity. None of the plots in S8-S23 suggest a correlation between the model parameters. All results below are based on a rejection algorithm using Euclidean distance. Using normalized root-mean-square-error produces nearly identical results, which can be found at https://github.com/rdinapoli/RN_demography.

Model 1

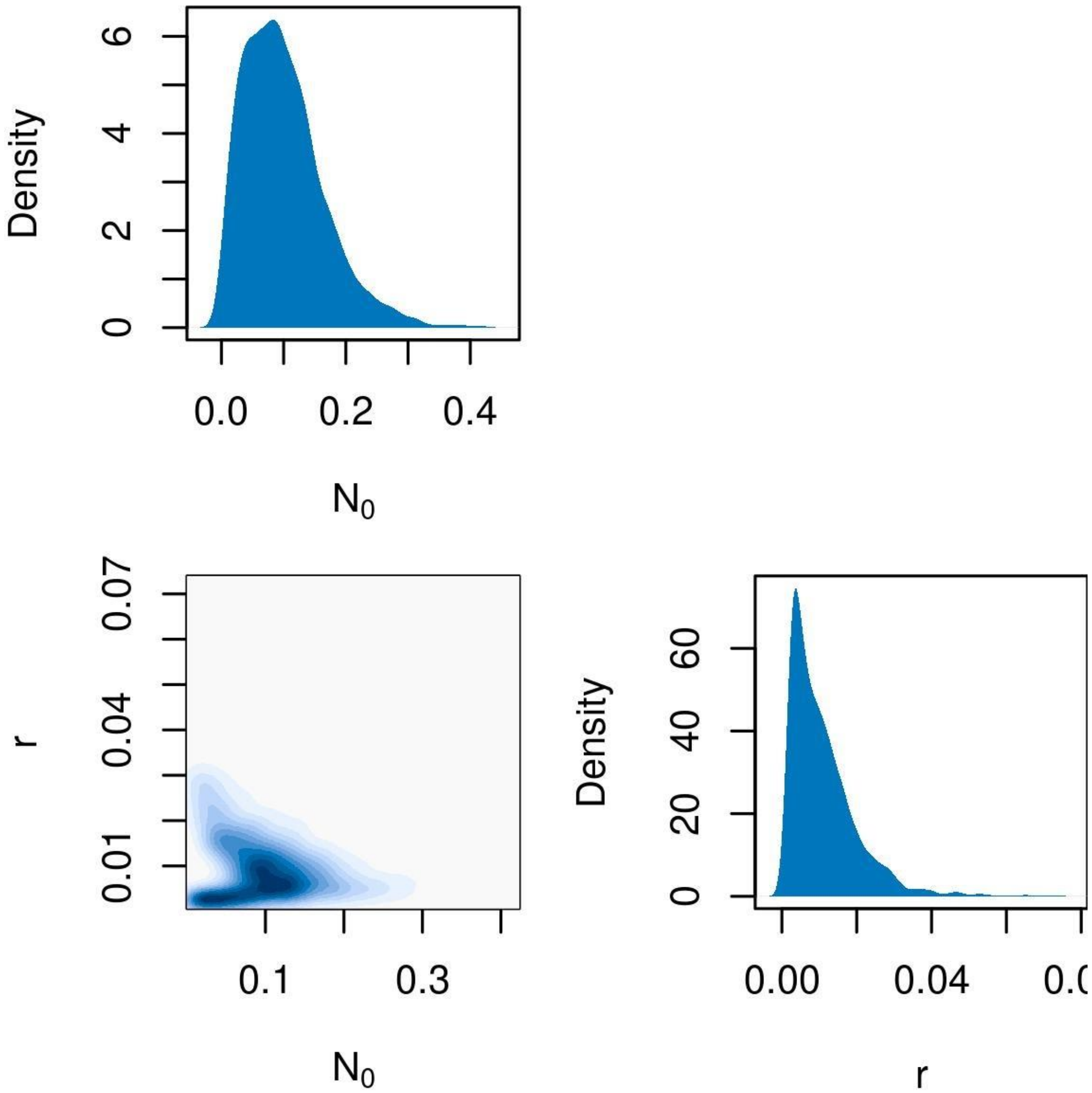

Figure S10. Model 1 joint posteriors using calsample and non-normalized SPD.

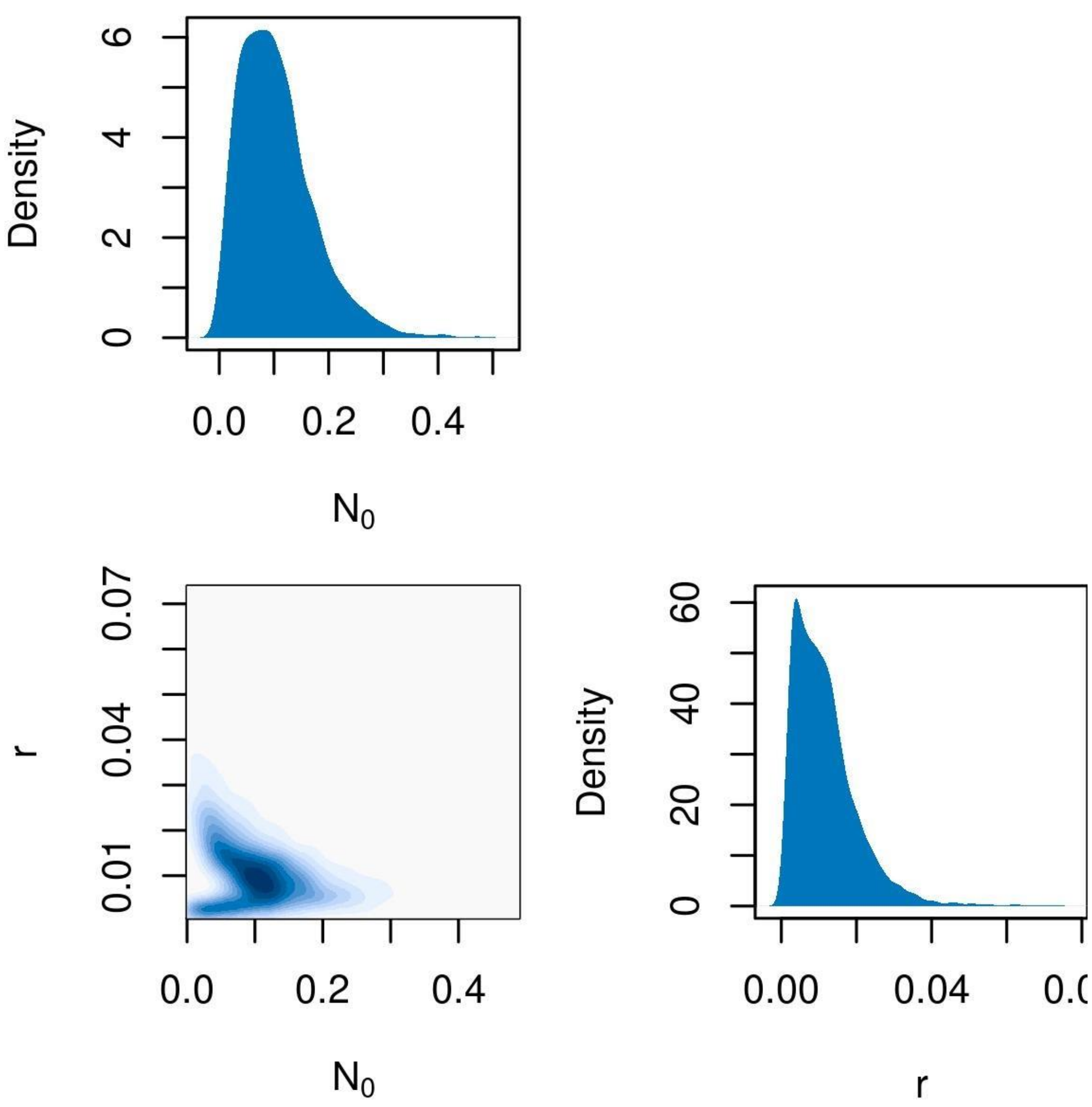

Figure S11. Model 1 joint posteriors using calsample and normalized SPD.

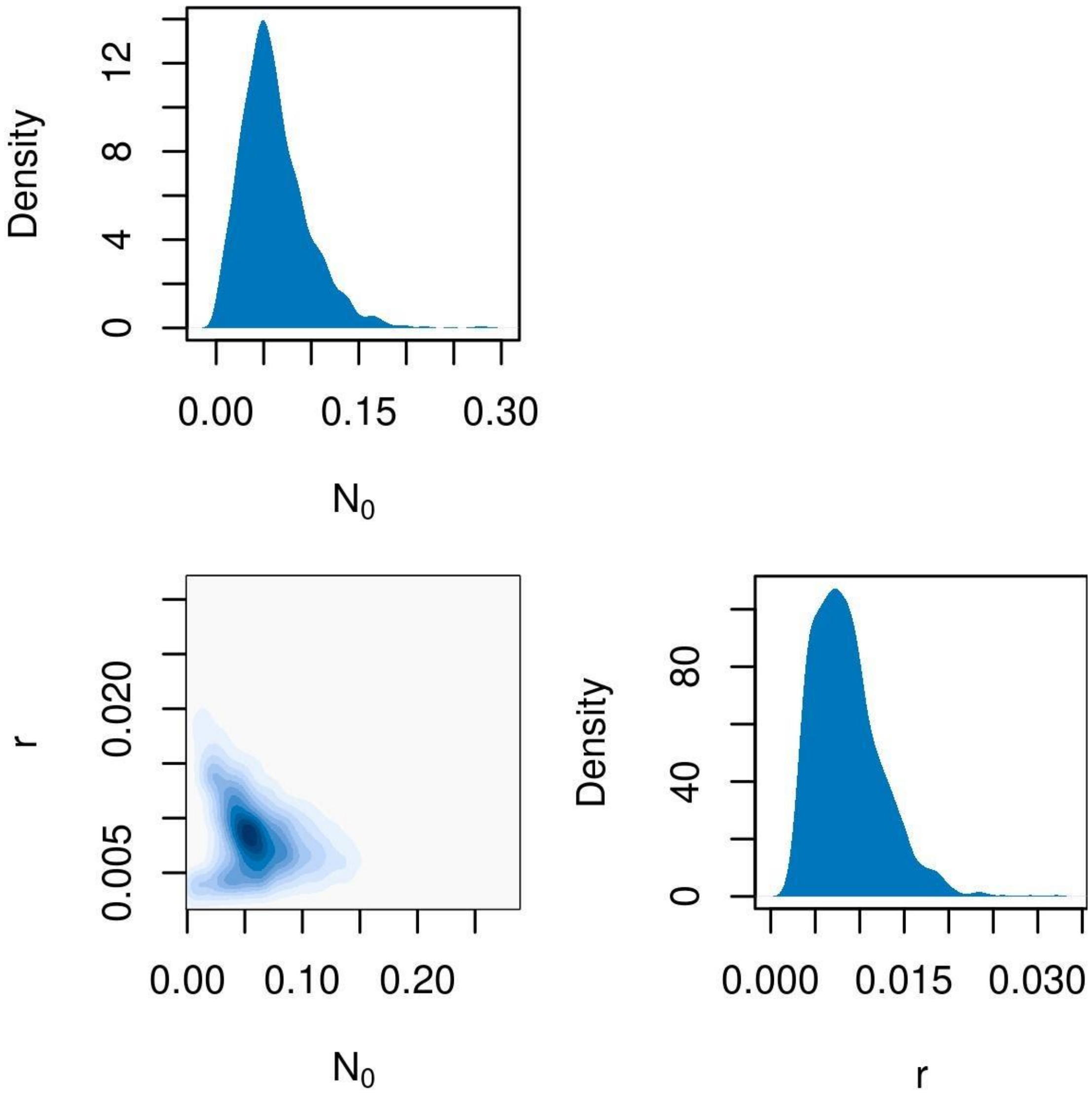

Figure S12. Model 1 joint posteriors using uncalsample and non-normalized SPD.

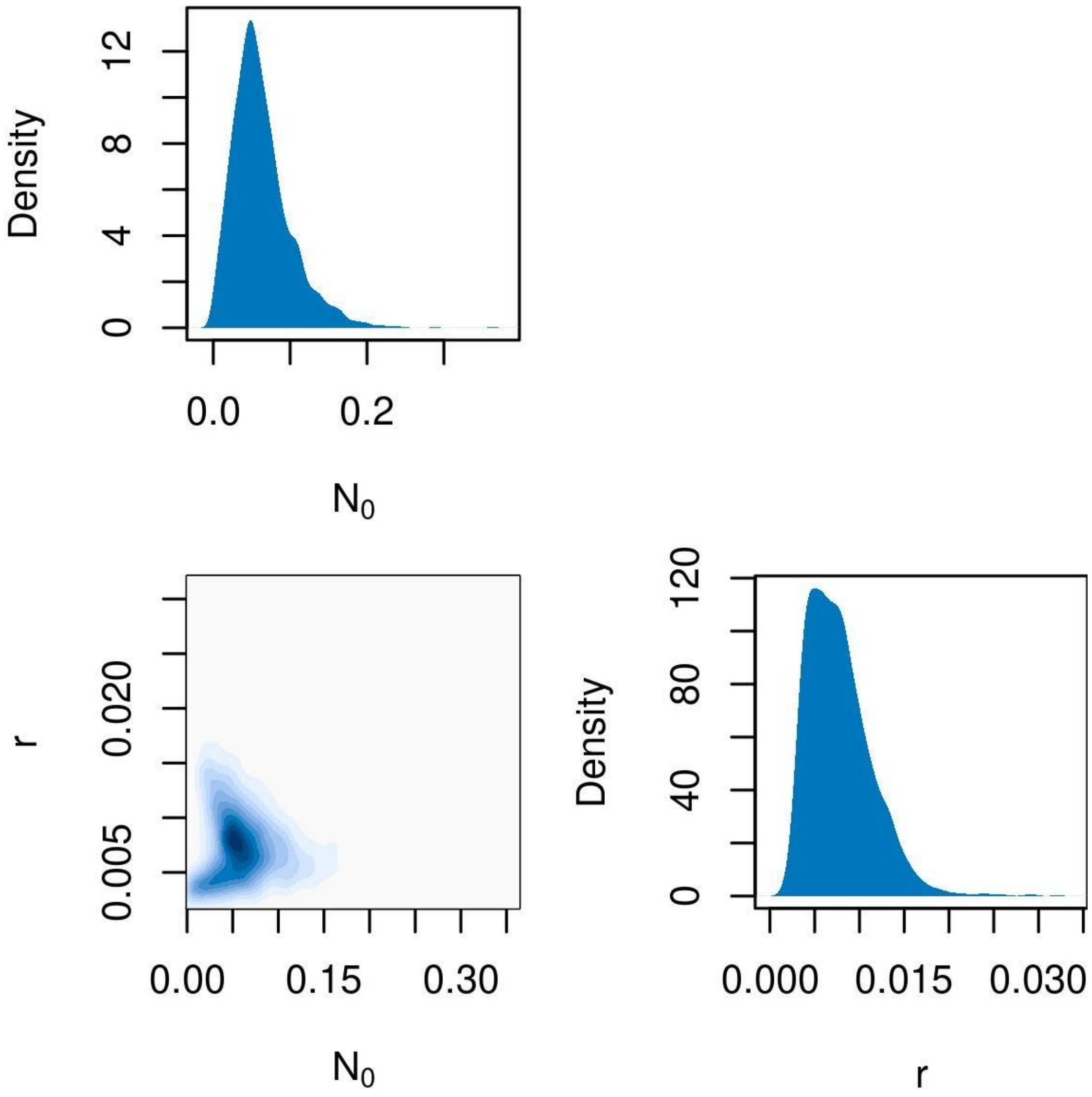

Figure S13. Model 1joint posteriors using uncalsample and normalized SPD.

Model 2

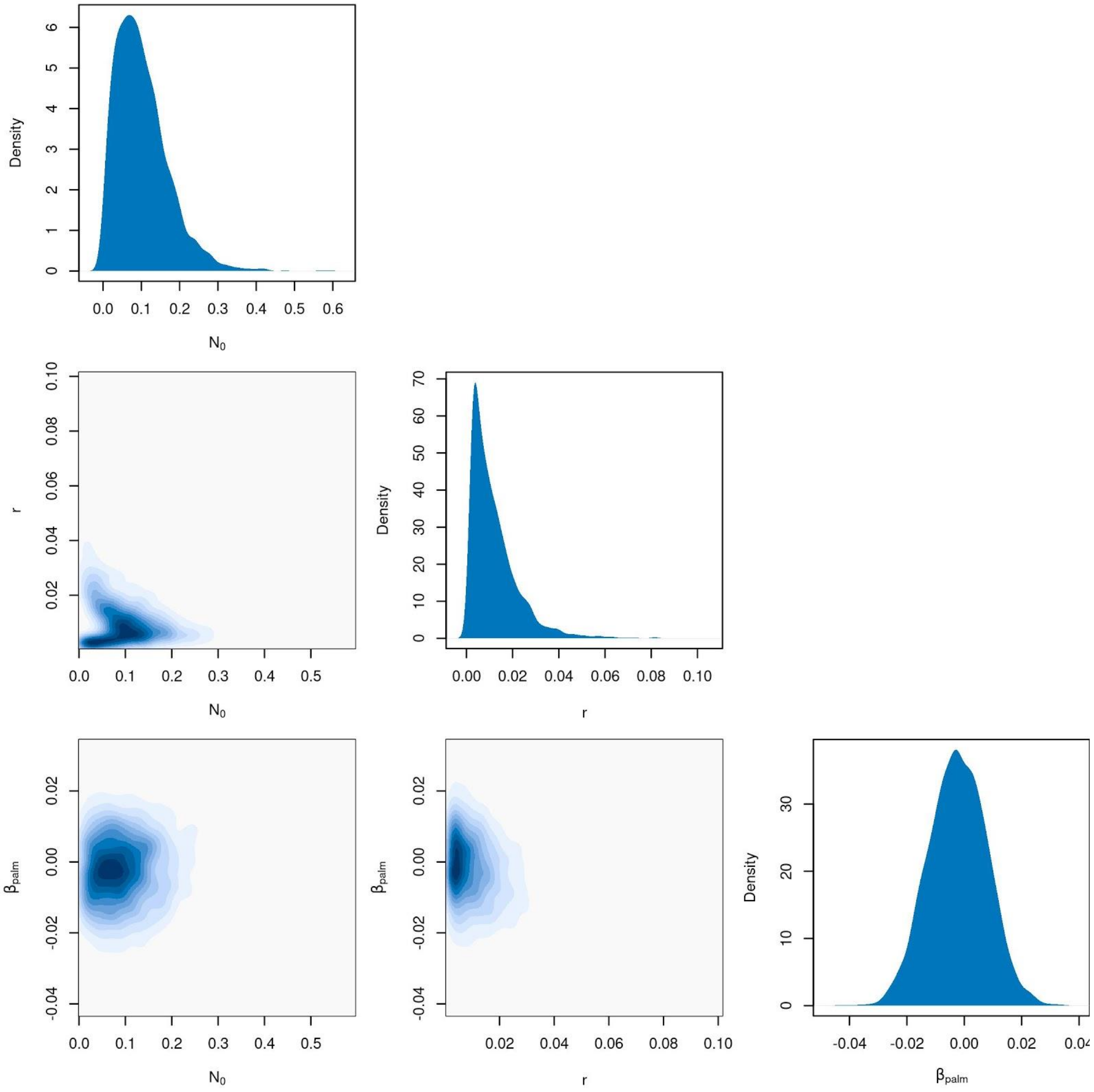

Figure S14. Model 2 joint posteriors using calsample and non-normalized SPD.

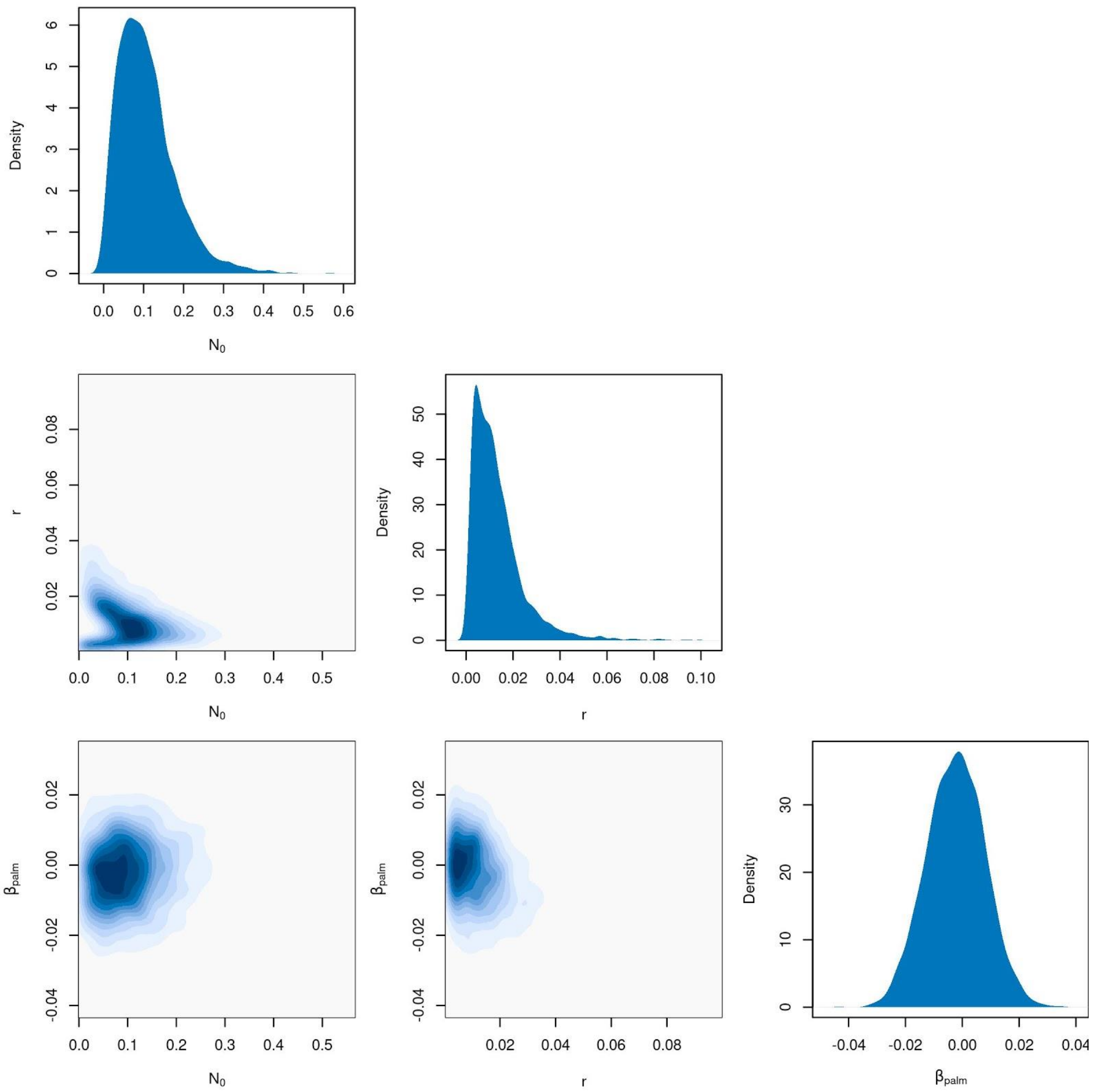

Figure S15. Model 2 joint posteriors using calsample and normalized SPD.

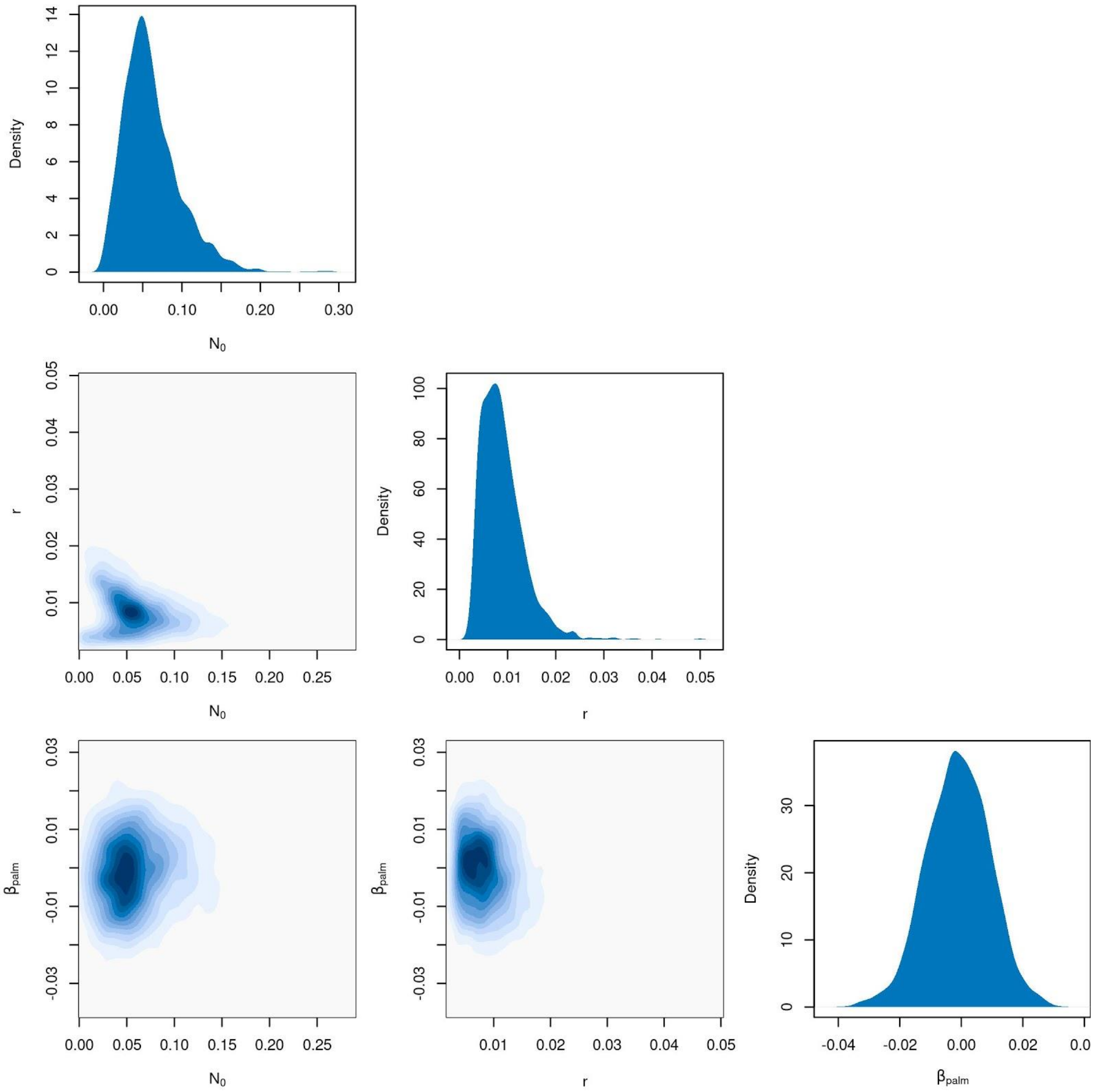

Figure S16. Model 2 joint posteriors using uncalsample and non-normalized SPD.

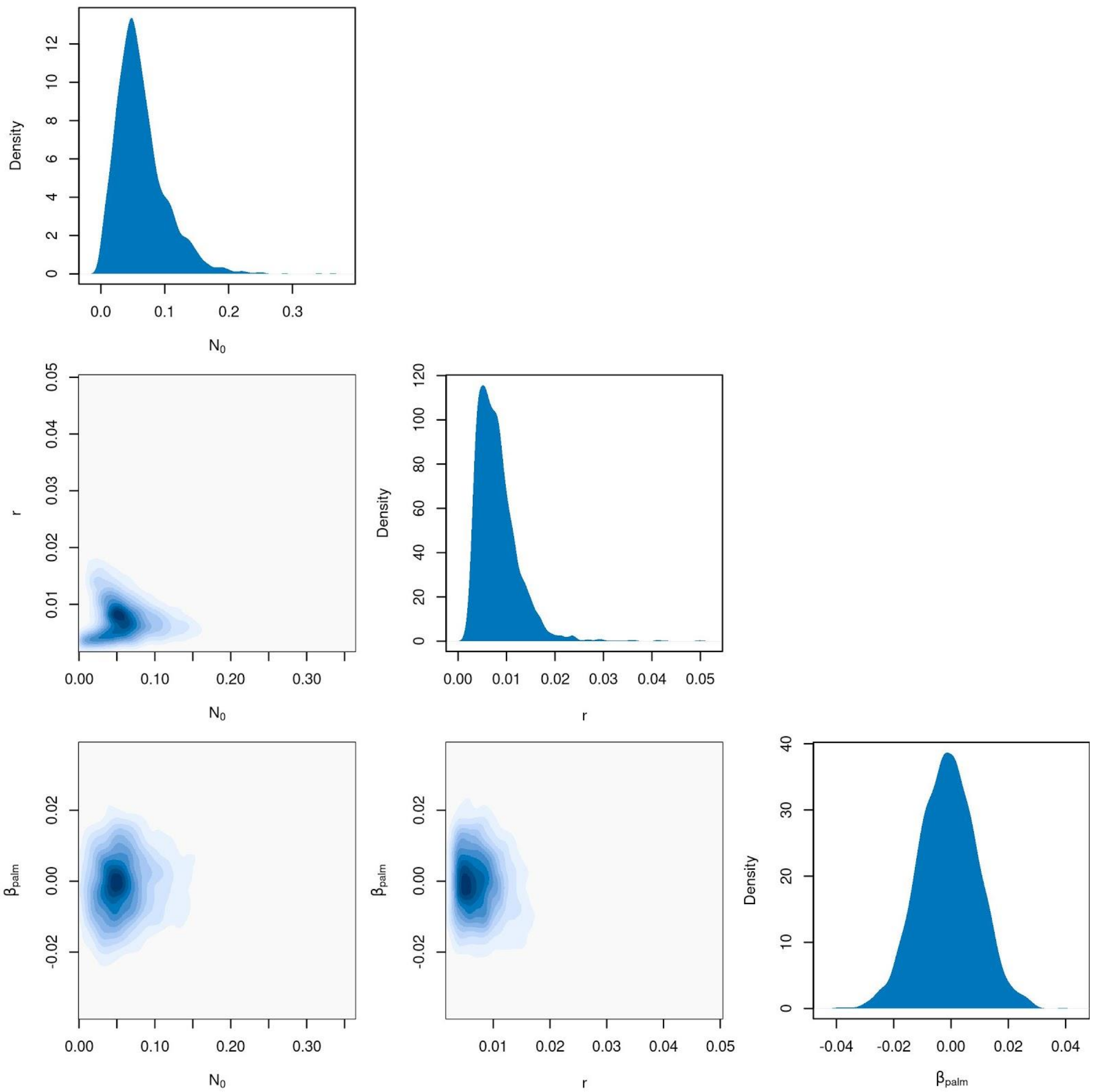

Figure S17. Model 2 joint posteriors using uncalsample and normalized SPD.

Model 3

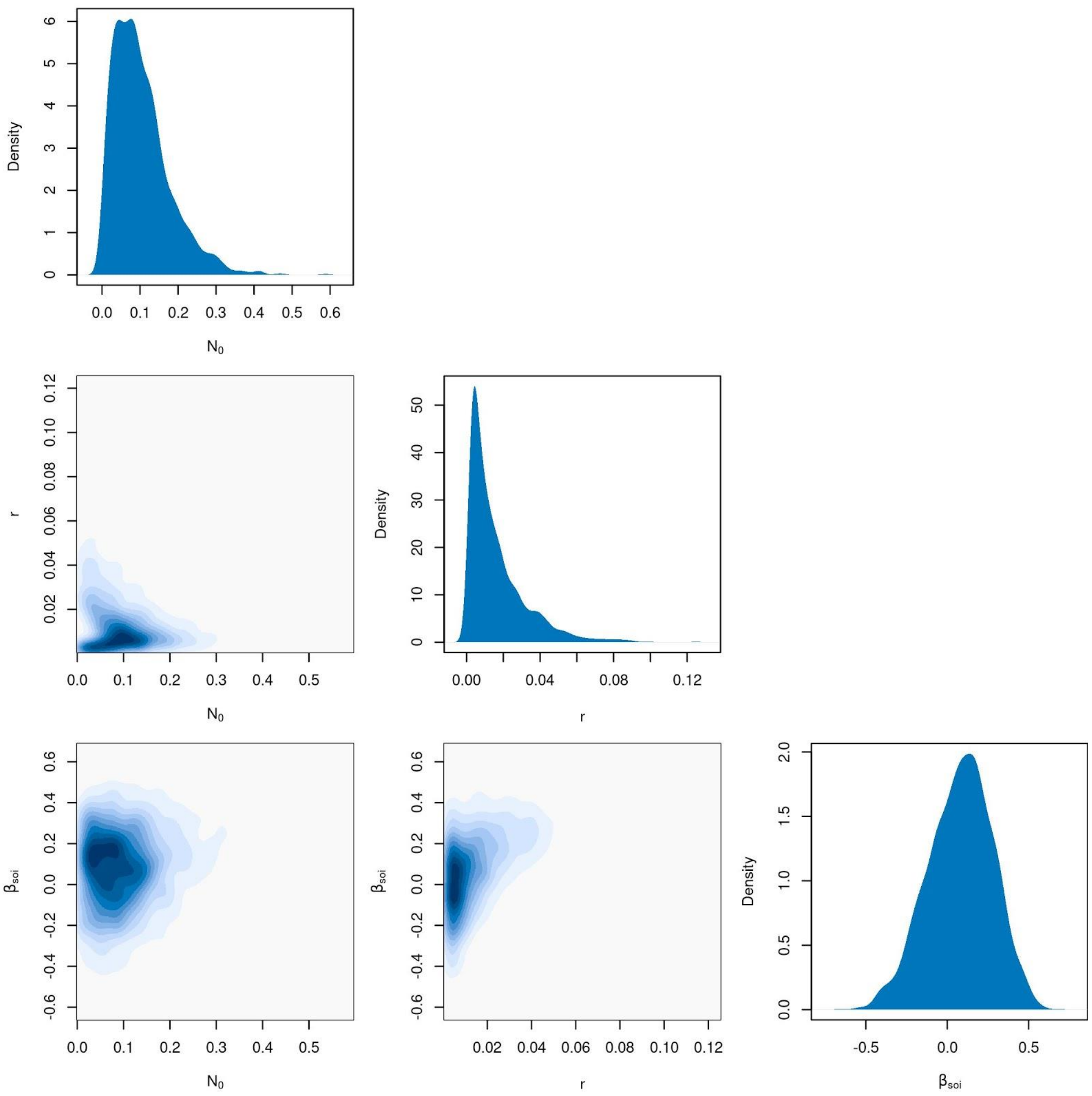

Figure S18. Model 3 joint posteriors using calsample and non-normalized SPD.

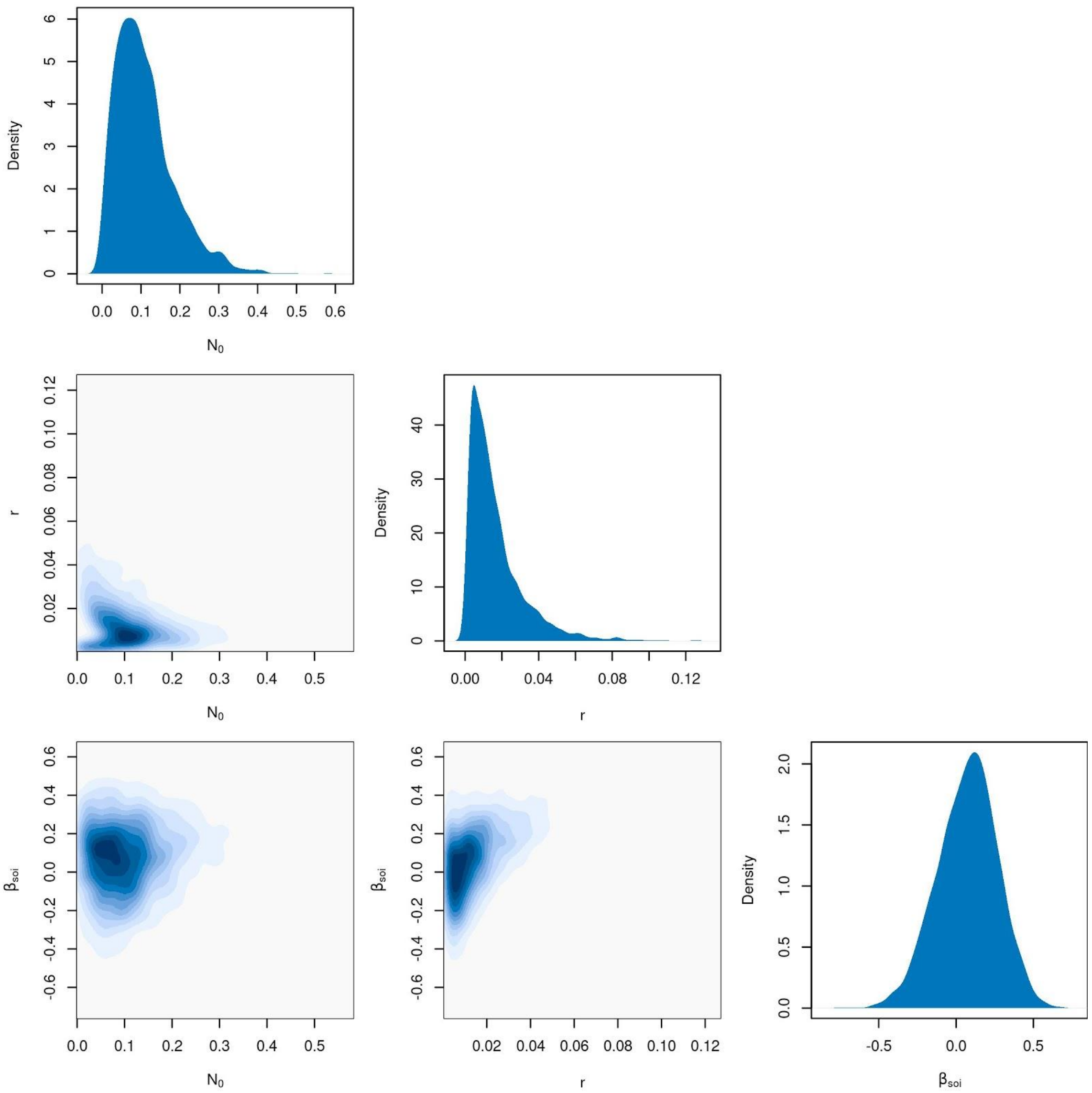

Figure S19. Model 3 joint posteriors using calsample and normalized SPD.

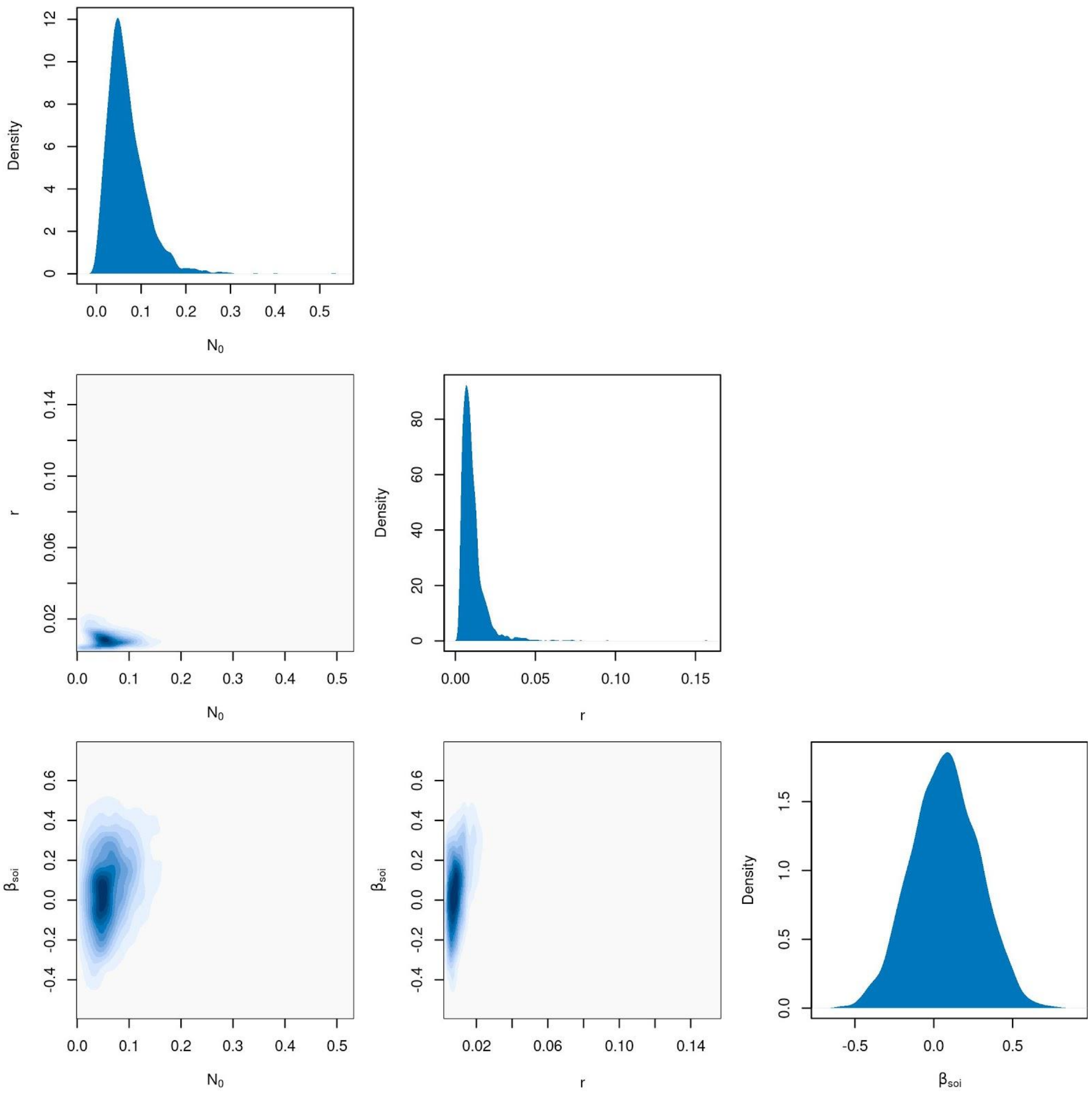

Figure S20. Model 3 joint posteriors using uncalsample and non-normalized SPD.

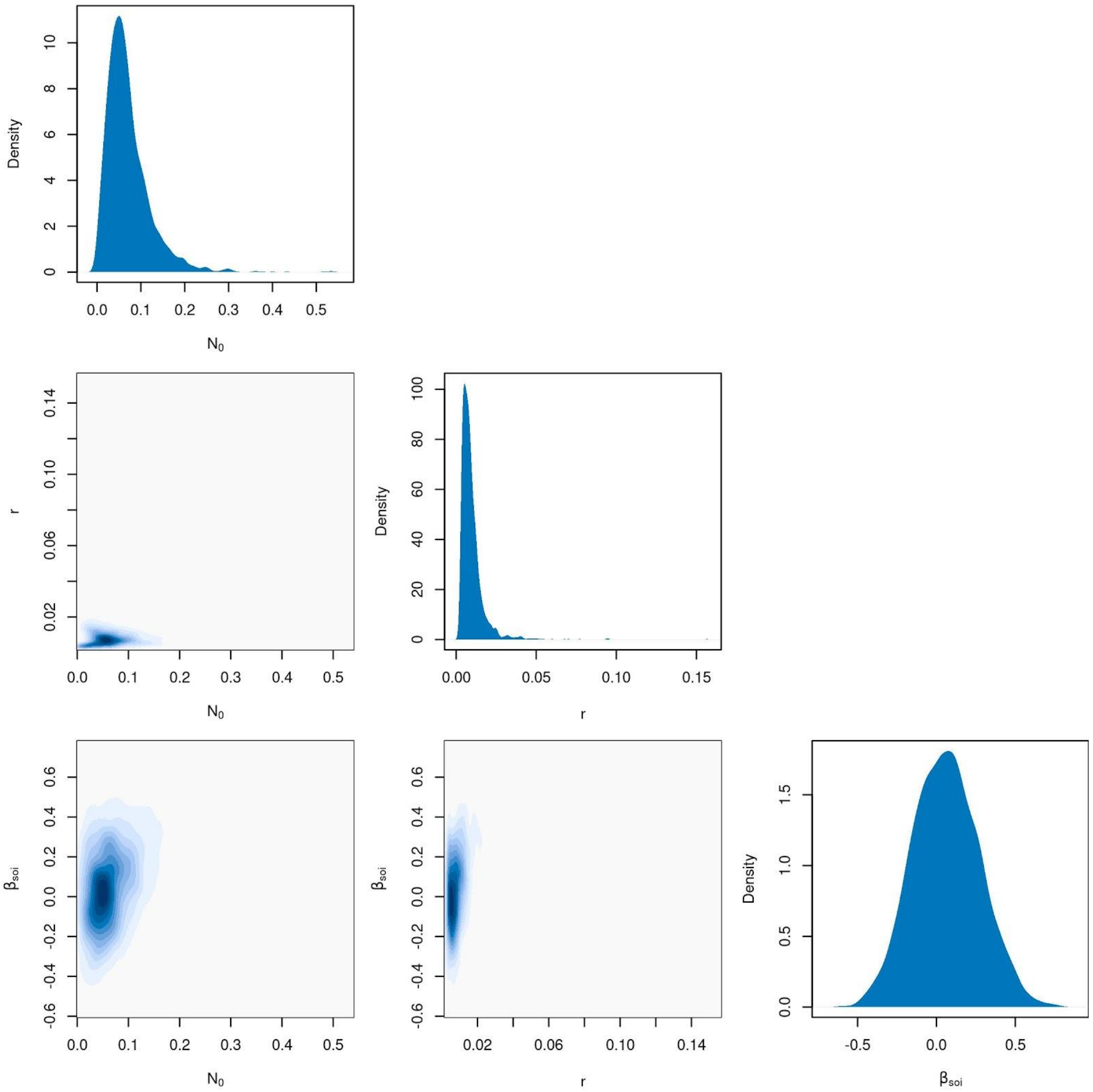

Figure S21. Model 3 joint posteriors using uncalsample and normalized SPD.

Model 4

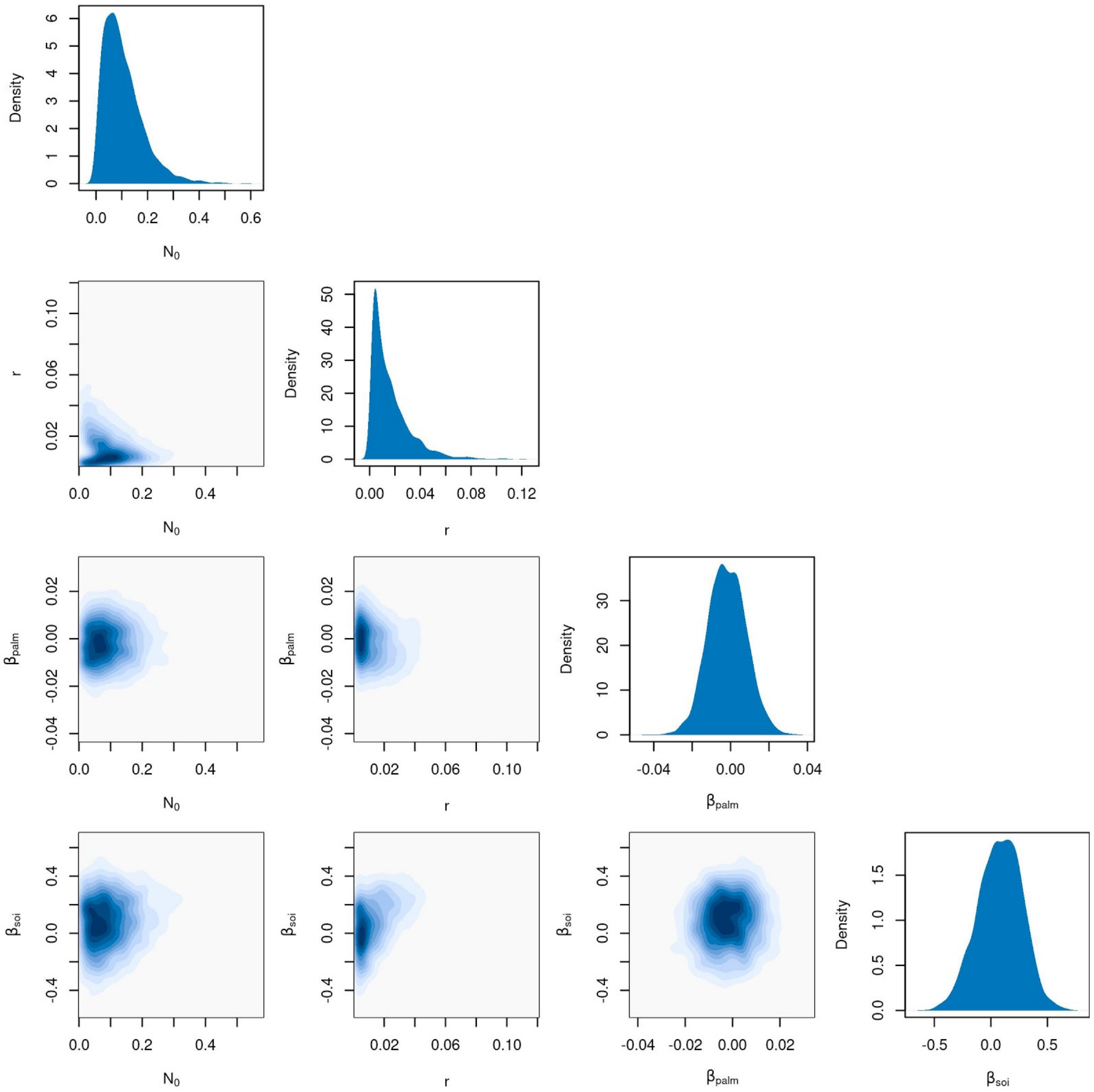

Figure S22. Model 4 joint posteriors using calsample and non-normalized SPD.

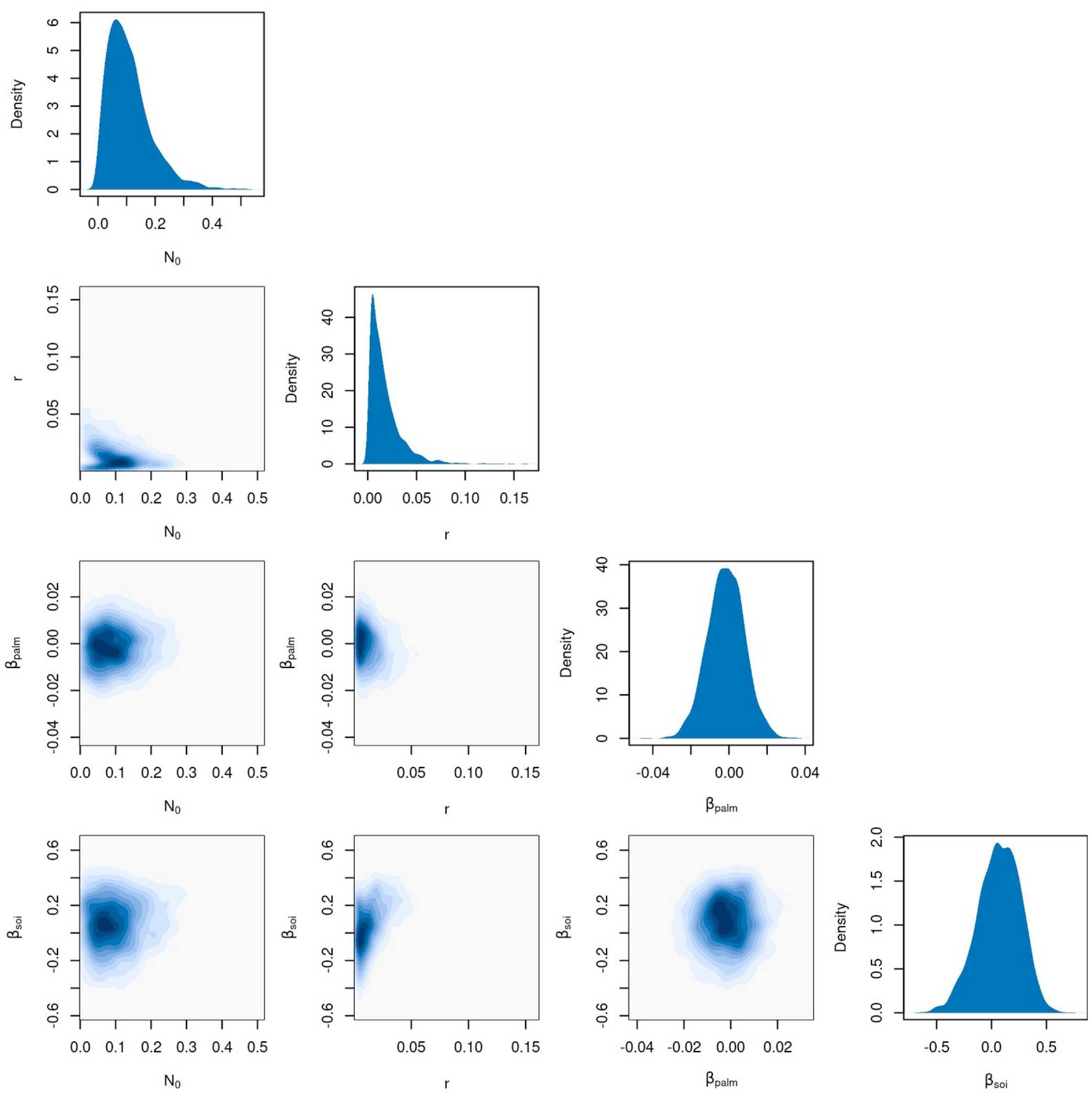

Figure S23. Model 4 joint posteriors using calsample and normalized SPD.

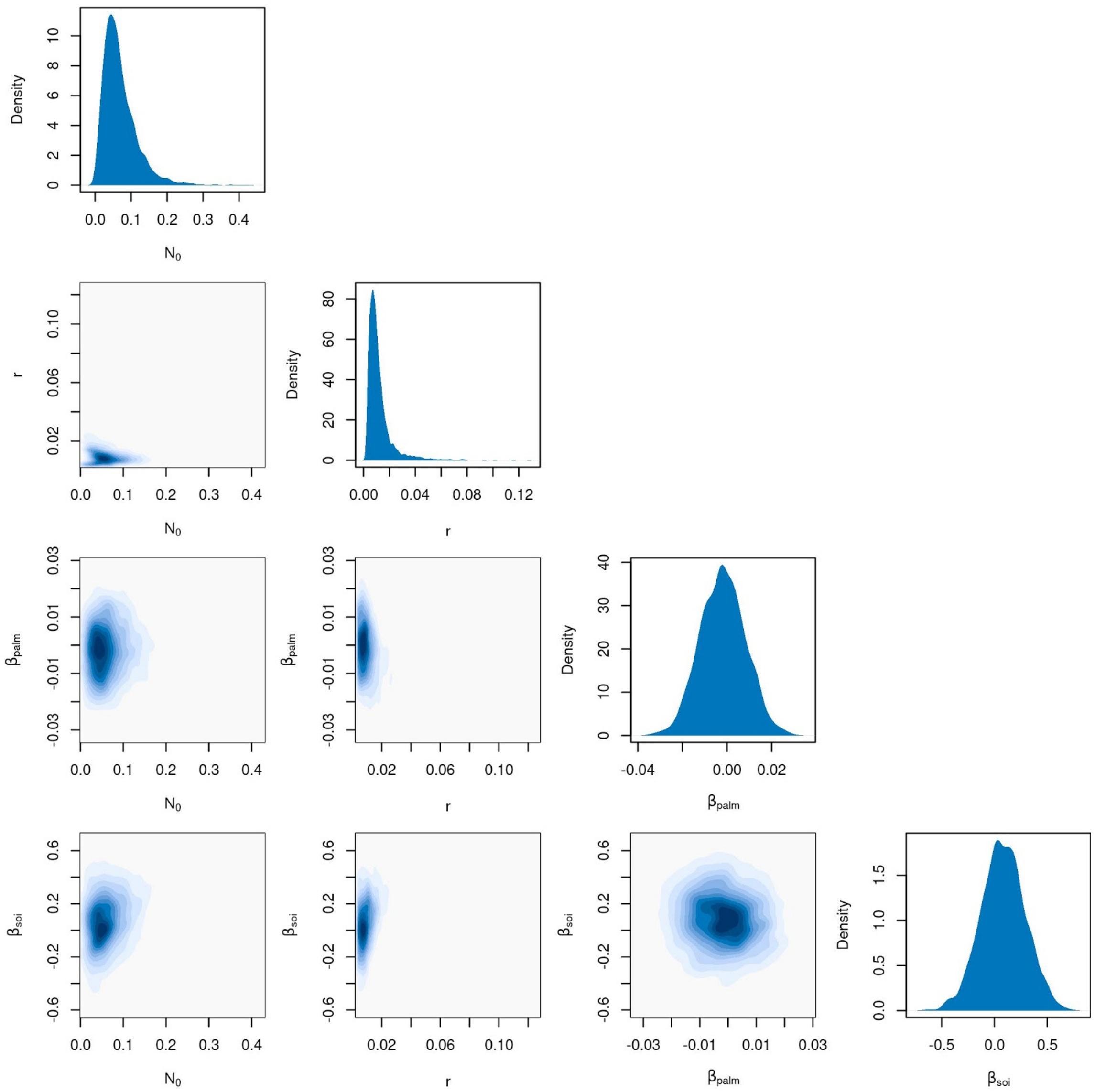

Figure S24. Model 4 joint posteriors using uncalsample and non-normalized SPD.

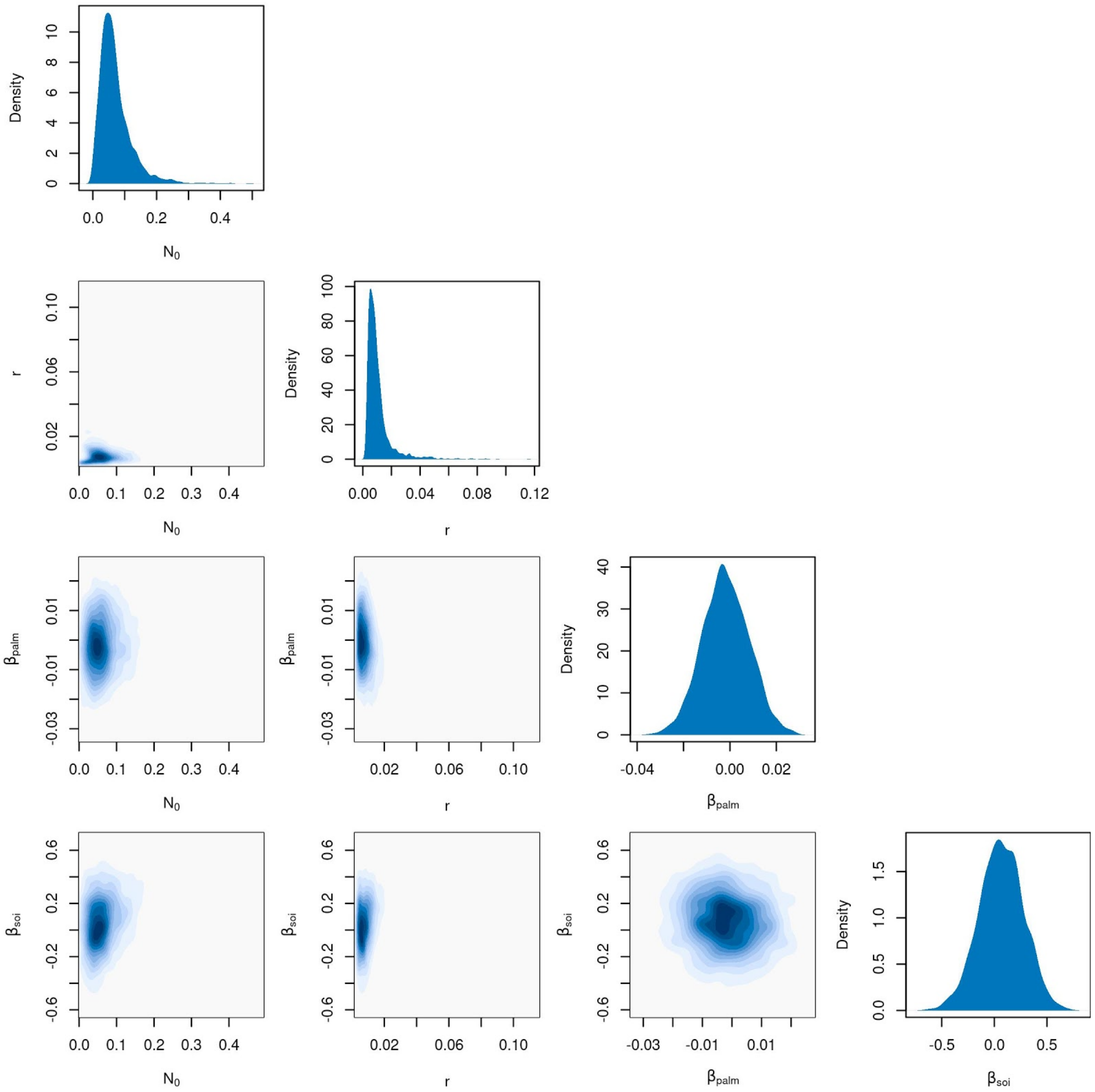

Figure S25. Model 4 joint posteriors using uncalsample and normalized SPD.

Supplementary Note 9: Marginal Posterior Distributions

Below, we present the marginal posterior distributions for each iteration of models 1-4. All results below (Fig. S24-S39) are based on a rejection algorithm using Euclidean distance. Using normalized root-mean-square-error produces nearly identical results, which can be found at https://github.com/rdinapoli/RN_demography.

Model 1

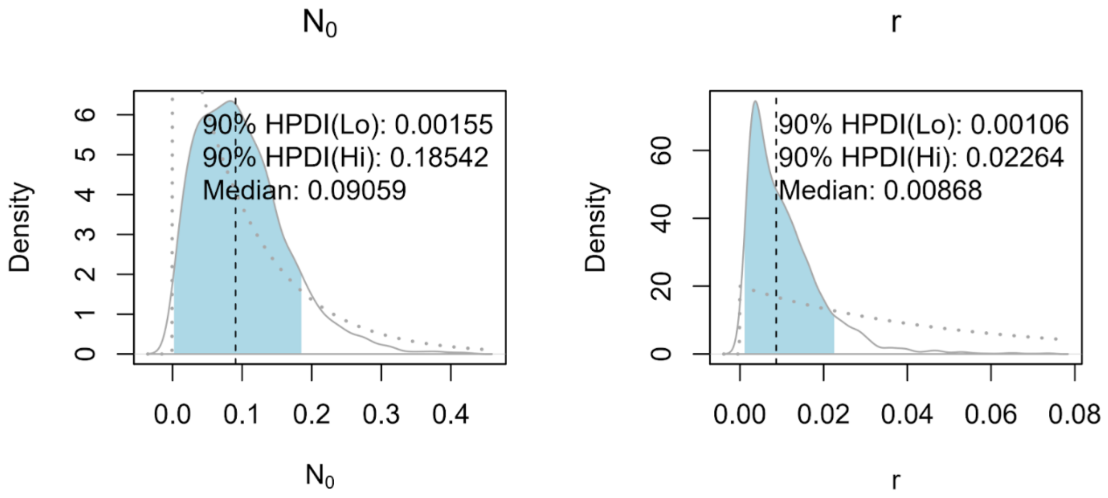

Figure S26. Model 1 posterior probabilities using calsample and non-normalized SPD. Dotted lines represent the prior distribution.

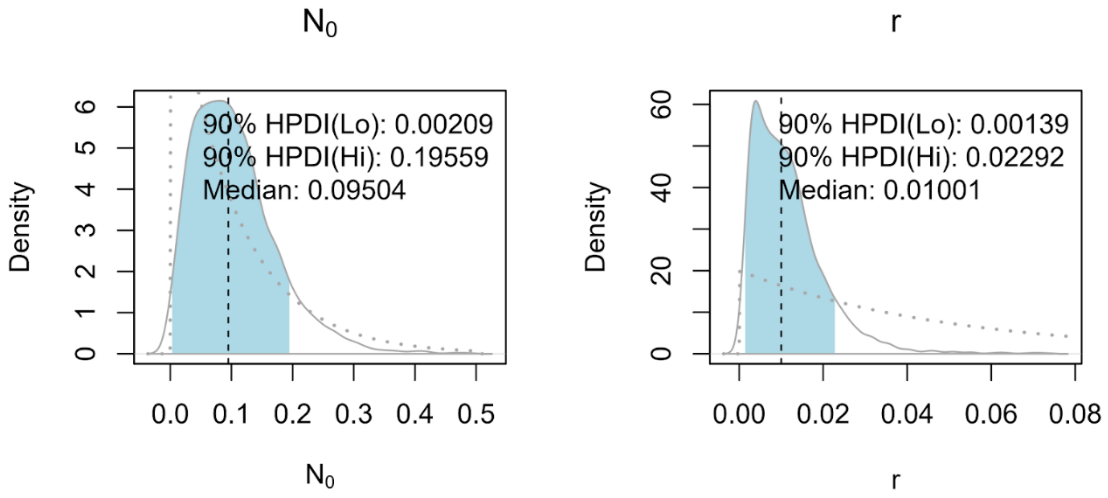

Figure S27. Model 1 posterior probabilities using calsample and normalized SPD. Dotted lines represent the prior distribution.

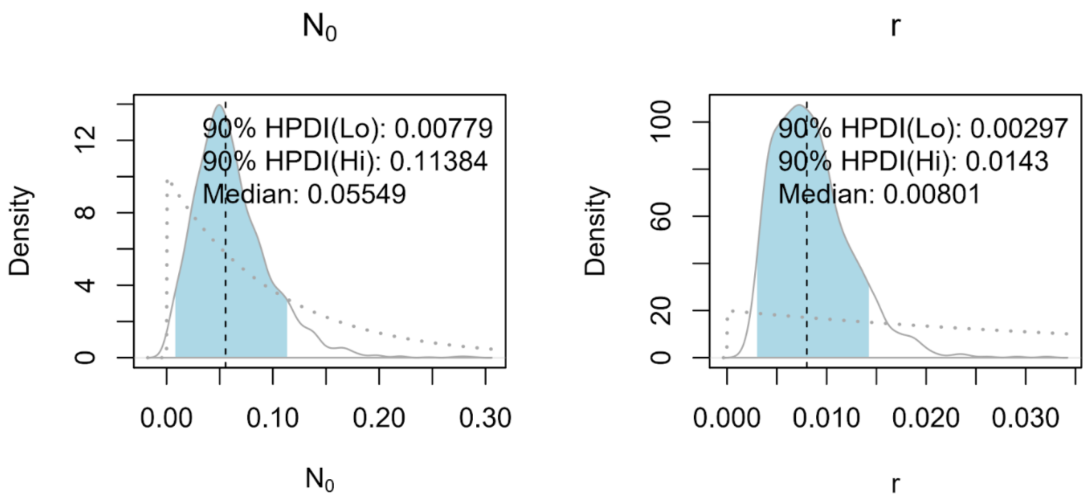

Figure S28. Model 1 posterior probabilities using uncalsample and non-normalized SPD. Note these are the results presented in the main text for model 1. Dotted lines represent the prior distribution.

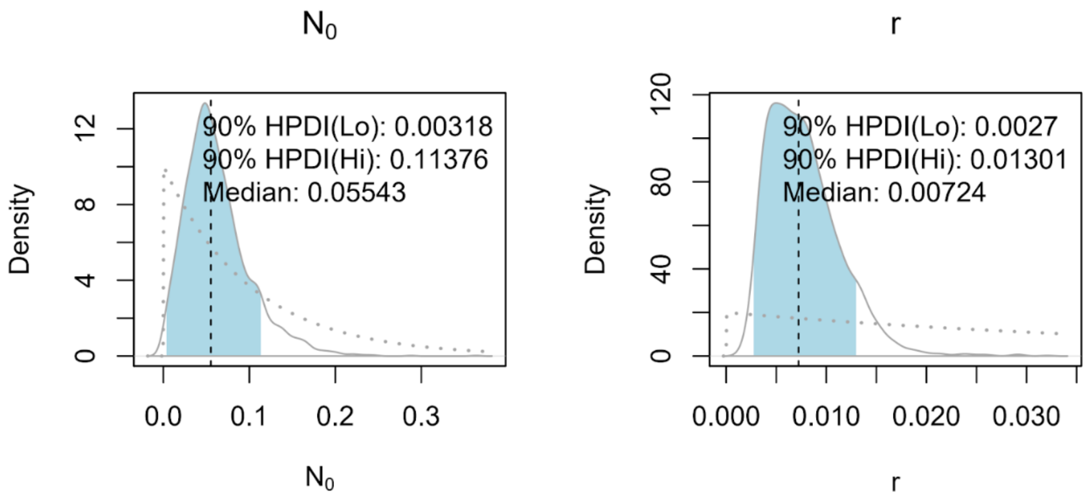

Figure S29. Model 1 posterior probabilities using uncalsample and normalized SPD. Dotted lines represent the prior distribution.

Model 2.

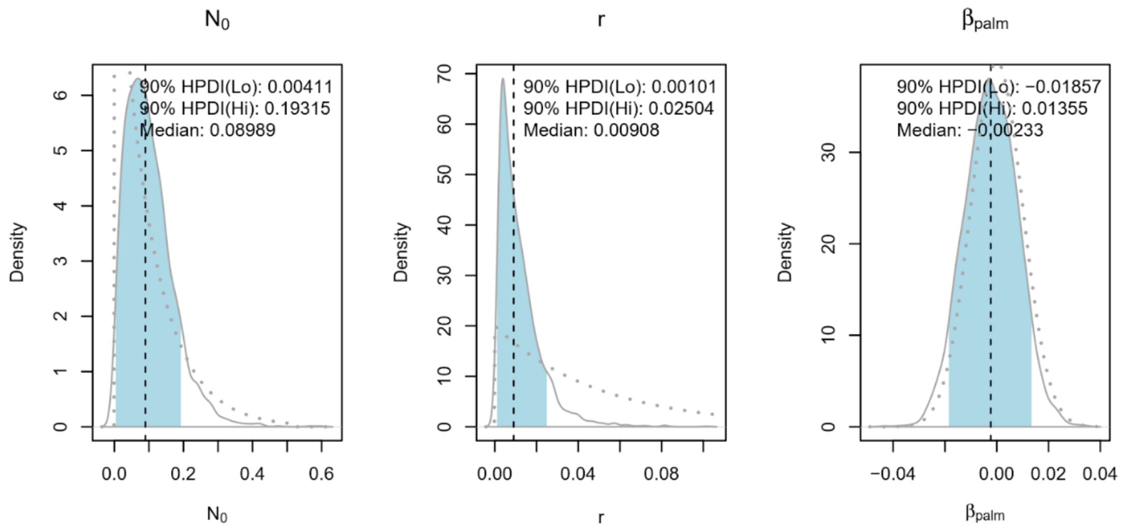

Figure S30. Model 2 posterior probabilities using calsample and non-normalized SPD. Dotted lines represent the prior distribution.

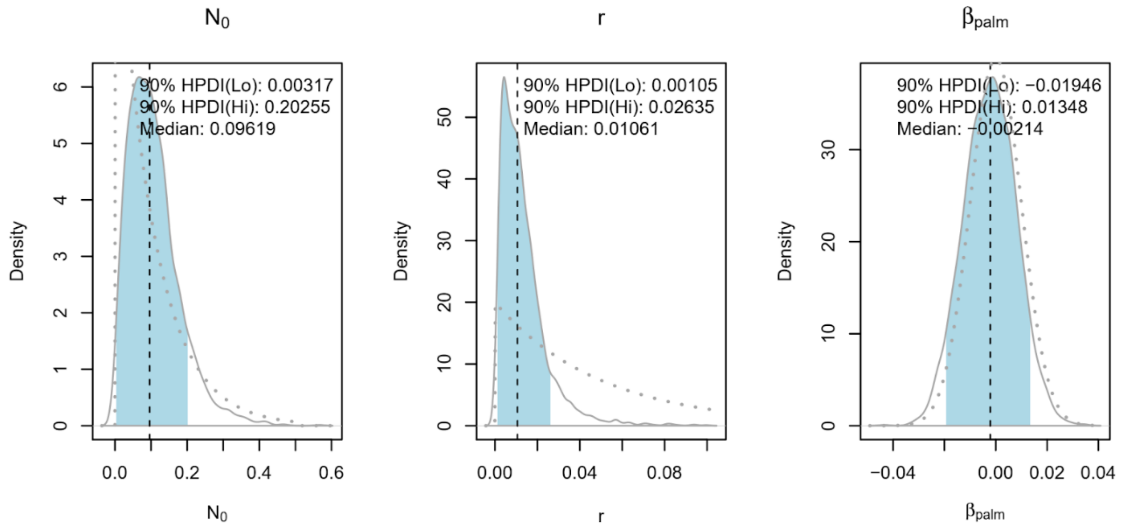

Figure S31. Model 2 posterior probabilities using calsample and normalized SPD. Dotted lines represent the prior distribution.

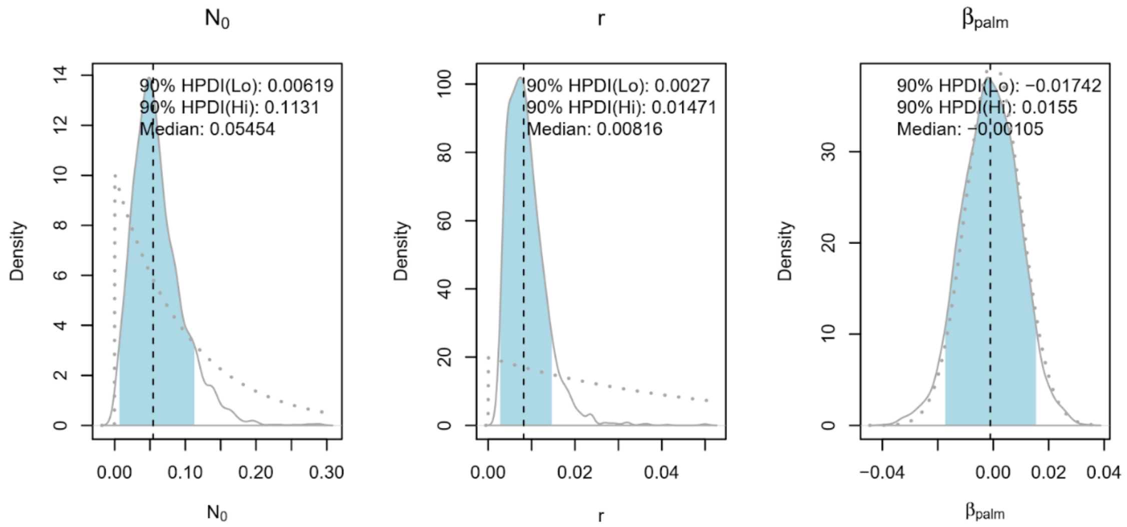

Figure S32. Model 2 posterior probabilities using uncalsample and non-normalized SPD. Note these are the results presented for Model 2 in the main text. Dotted lines represent the prior distribution.

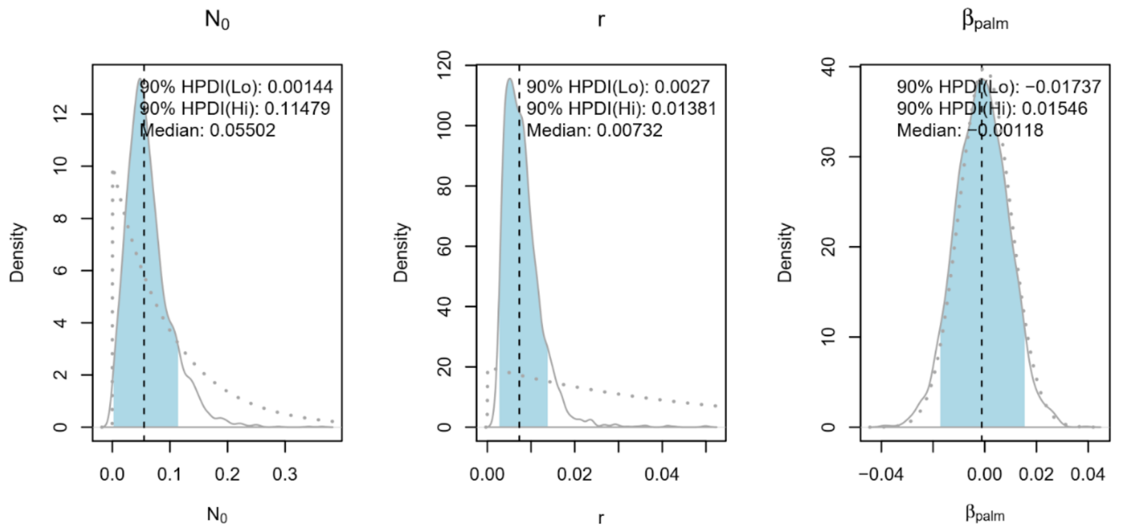

Figure S33. Model 2 posterior probabilities using uncalsample and normalized SPD. Dotted lines represent the prior distribution.

Model 3

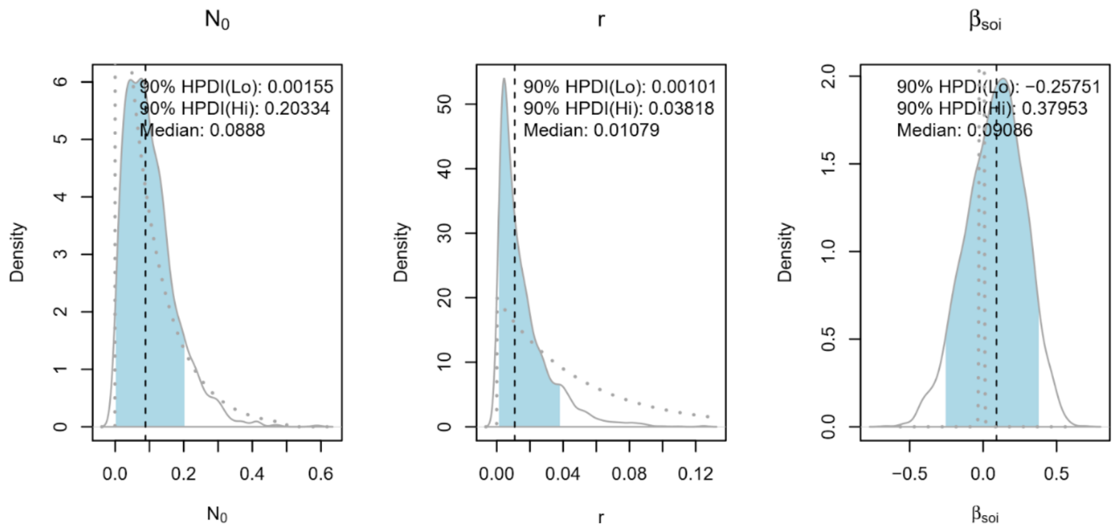

Figure S34. Model 3 posterior probabilities using calsample and non-normalized SPD. Dotted lines represent the prior distribution.

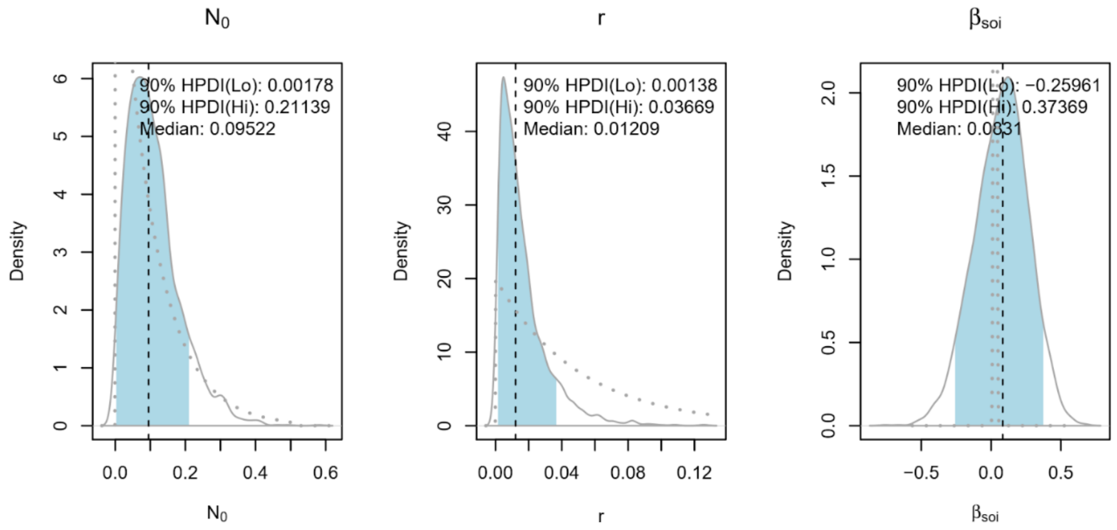

Figure S35. Model 3 posterior probabilities using calsample and normalized SPD. Dotted lines represent the prior distribution.

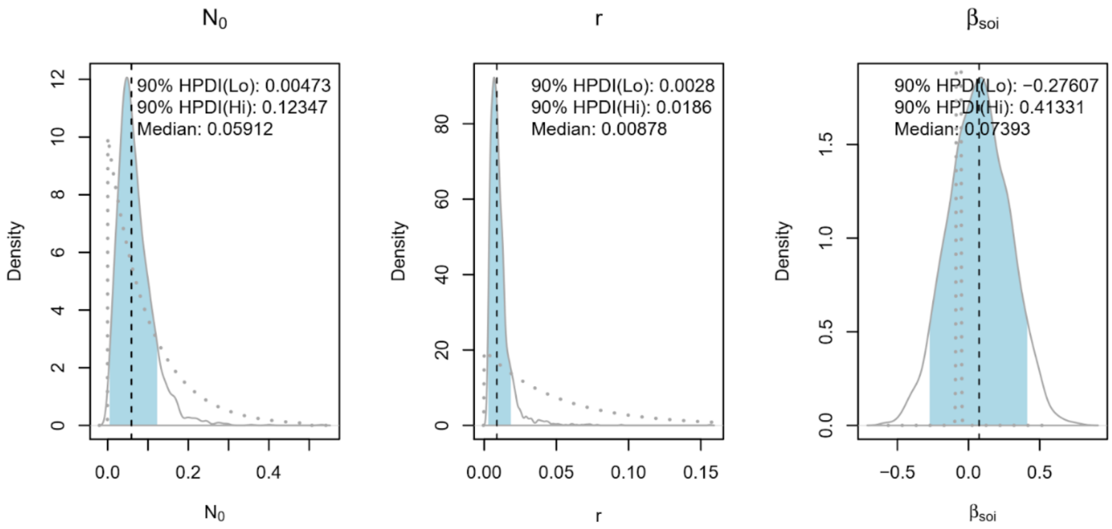

Figure S36. Model 3 posterior probabilities using uncalsample and non-normalized SPD. Note these are the results presented for Model 3 in the main text. Dotted lines represent the prior distribution.

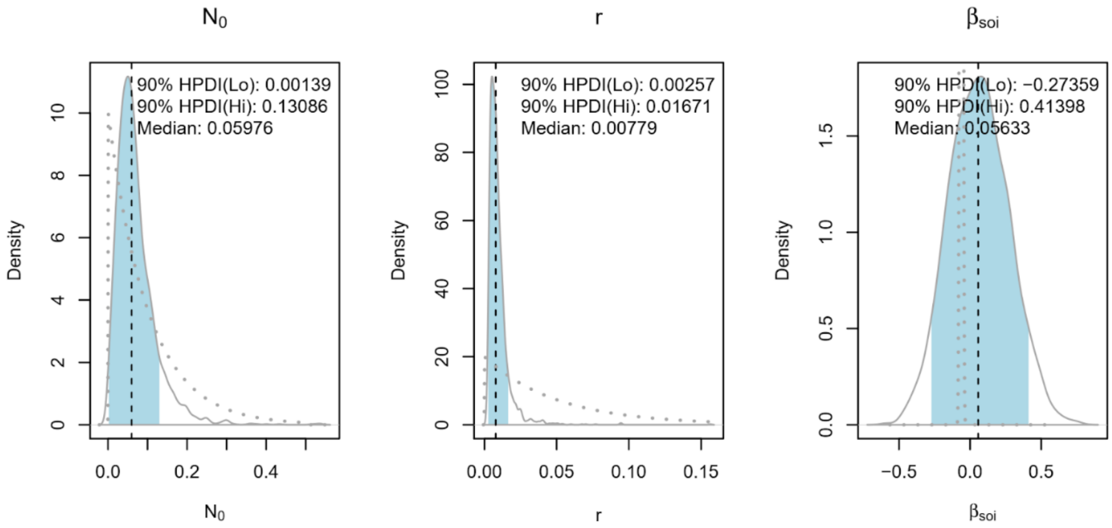

Figure S37. Model 3 posterior probabilities using uncalsample and normalized SPD. Dotted lines represent the prior distribution.

Model 4.

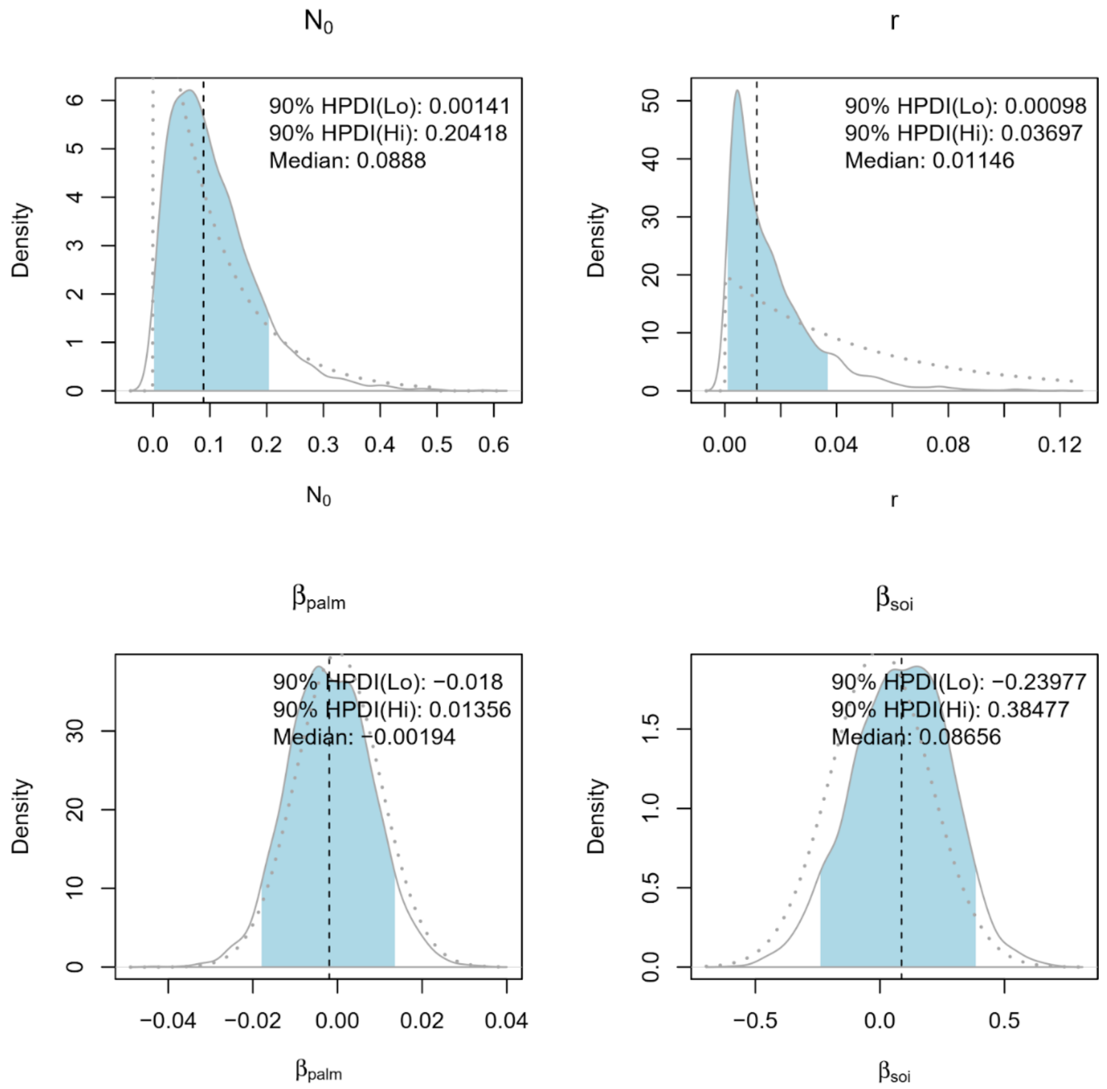

Figure S38. Model 4 posterior probabilities using calsample and non-normalized SPD. Dotted lines represent the prior distribution.

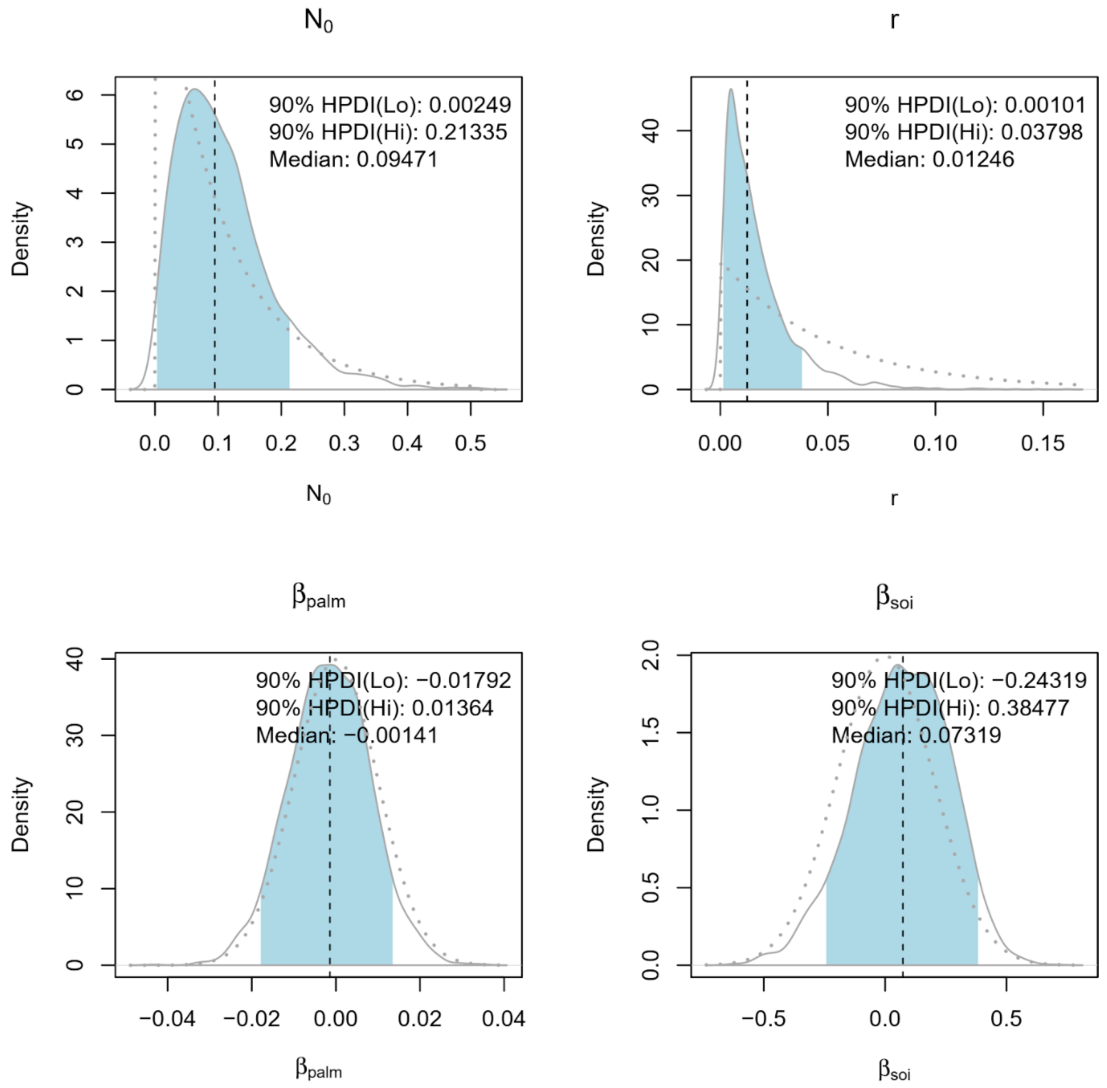

Figure S39. Model 4 posterior probabilities using calsample and normalized SPD. Dotted lines represent the prior distribution.

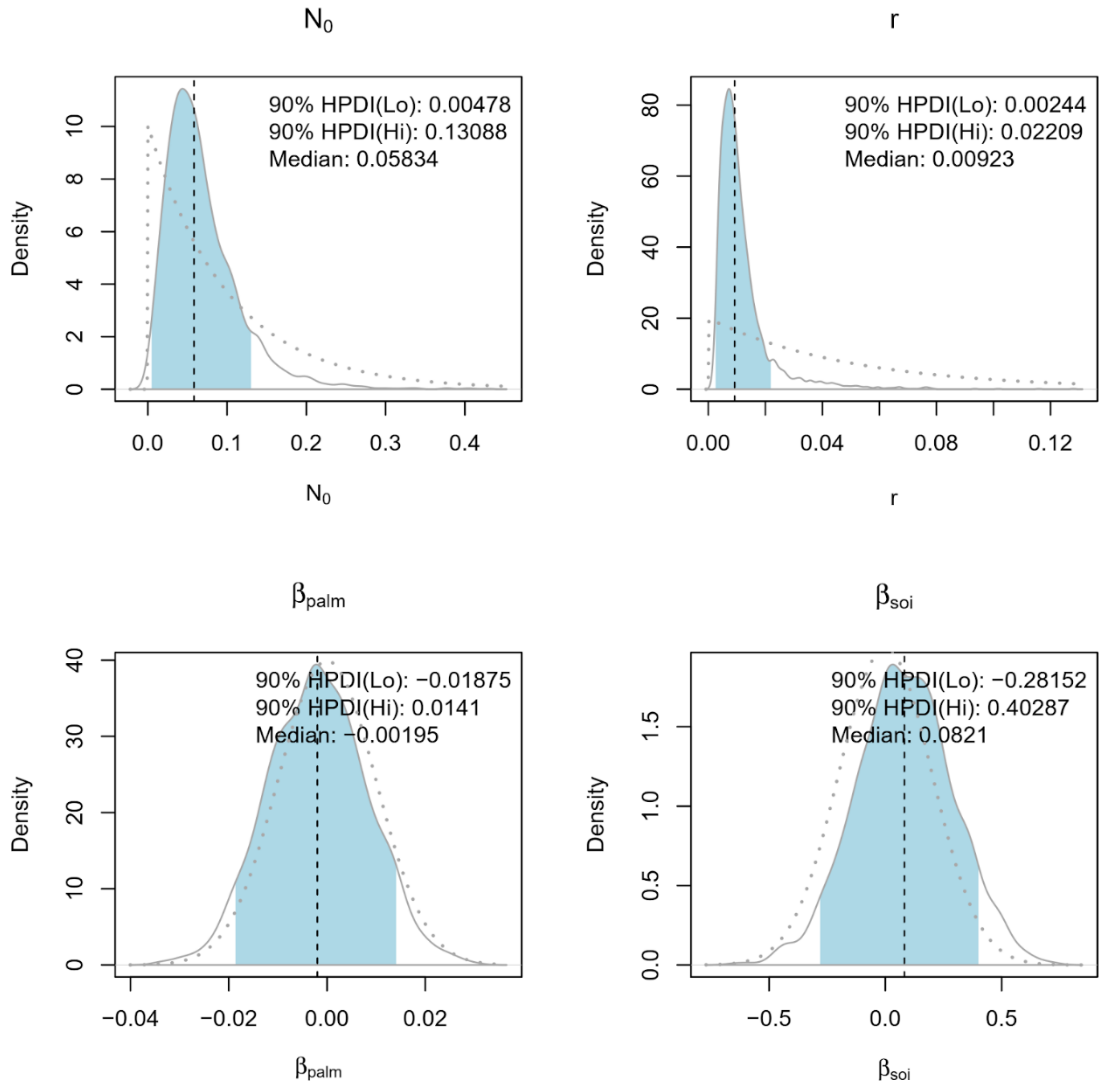

Figure S40. Model 4 posterior probabilities using uncalsample and non-normalized SPD. Note that these are the results presented for Model 4 in the main text. Dotted lines represent the prior distribution.

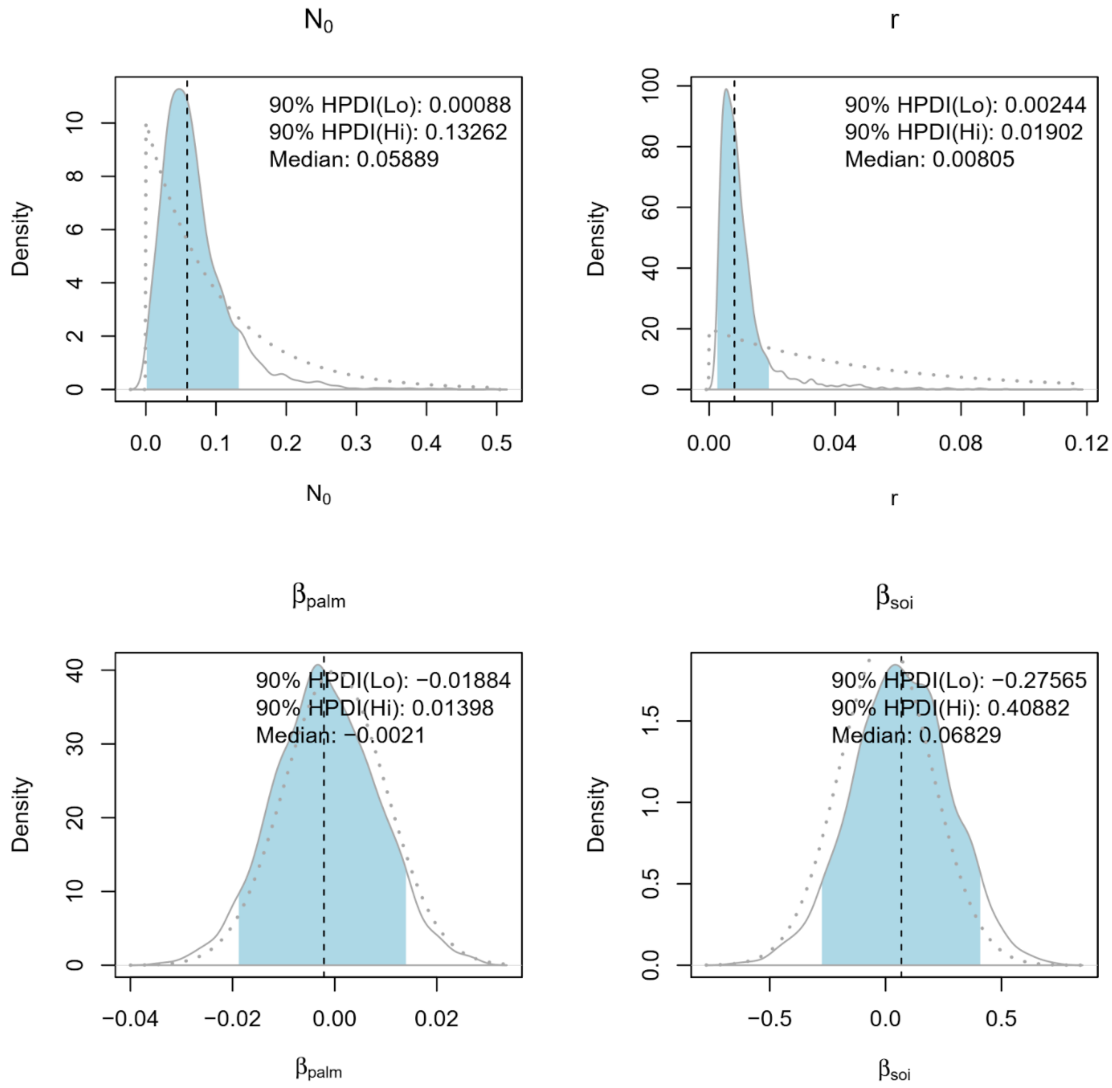

Figure S41. Model 4 posterior probabilities using uncalsample and normalized SPD.

Supplementary Note 10: SPD Posterior Predictive Checks

The plots below show posterior predictive checks (PPC) of the observed Rapa Nui SPD fitted to the four demographic models for this portion of the Southern Hemisphere radiocarbon calibration curve [8]. All results (S40-S43) below are based on a rejection algorithm using Euclidean distance. Using normalized root-mean-square-error produces nearly identical results, which can be found at https://github.com/rdinapoli/RN_demography

Model 1

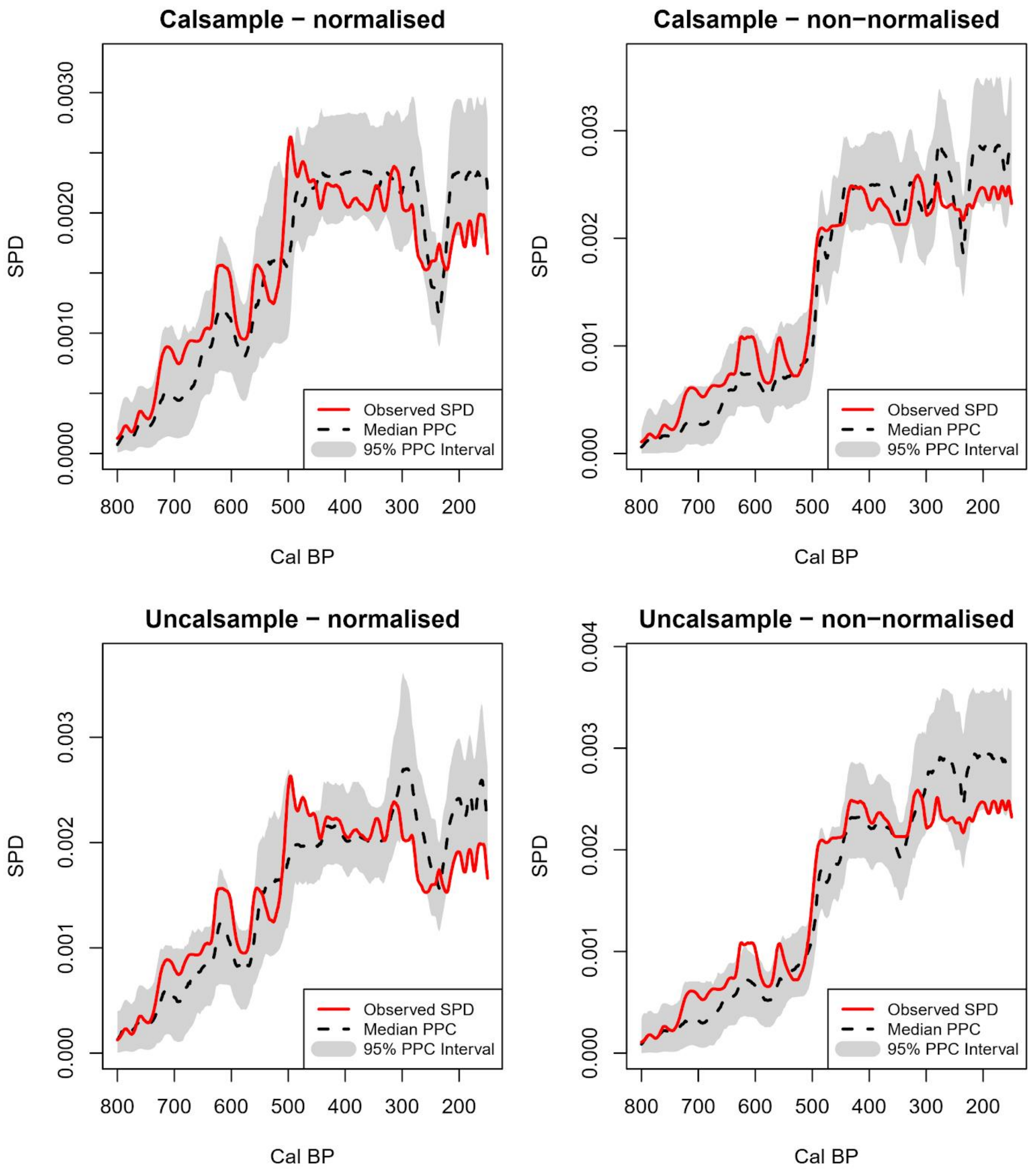

Figure S42. Posterior results for model 1 using four combinations of normalization and back-calibration. The solid red line is the observed SPD, the dashed black line is the median of the PCC, and the grey shading shows the 95% PPC interval.

Model 2

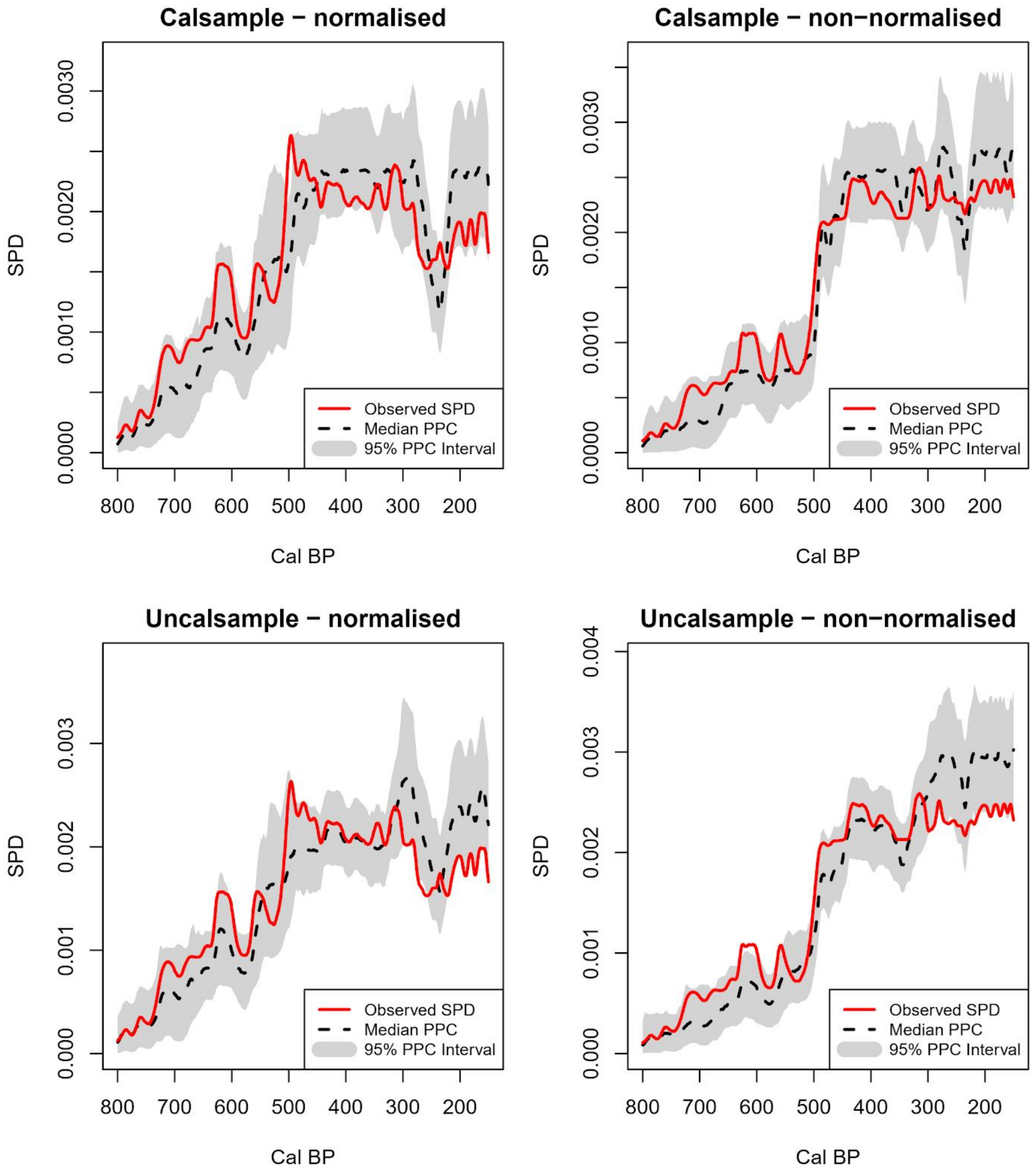

Figure S43. Posterior results for model 2 using four combinations of normalization and back-calibration. The solid red line is the observed SPD, the dashed black line is the median of the PCC, and the grey shading shows the 95% PPC interval.

Model 3

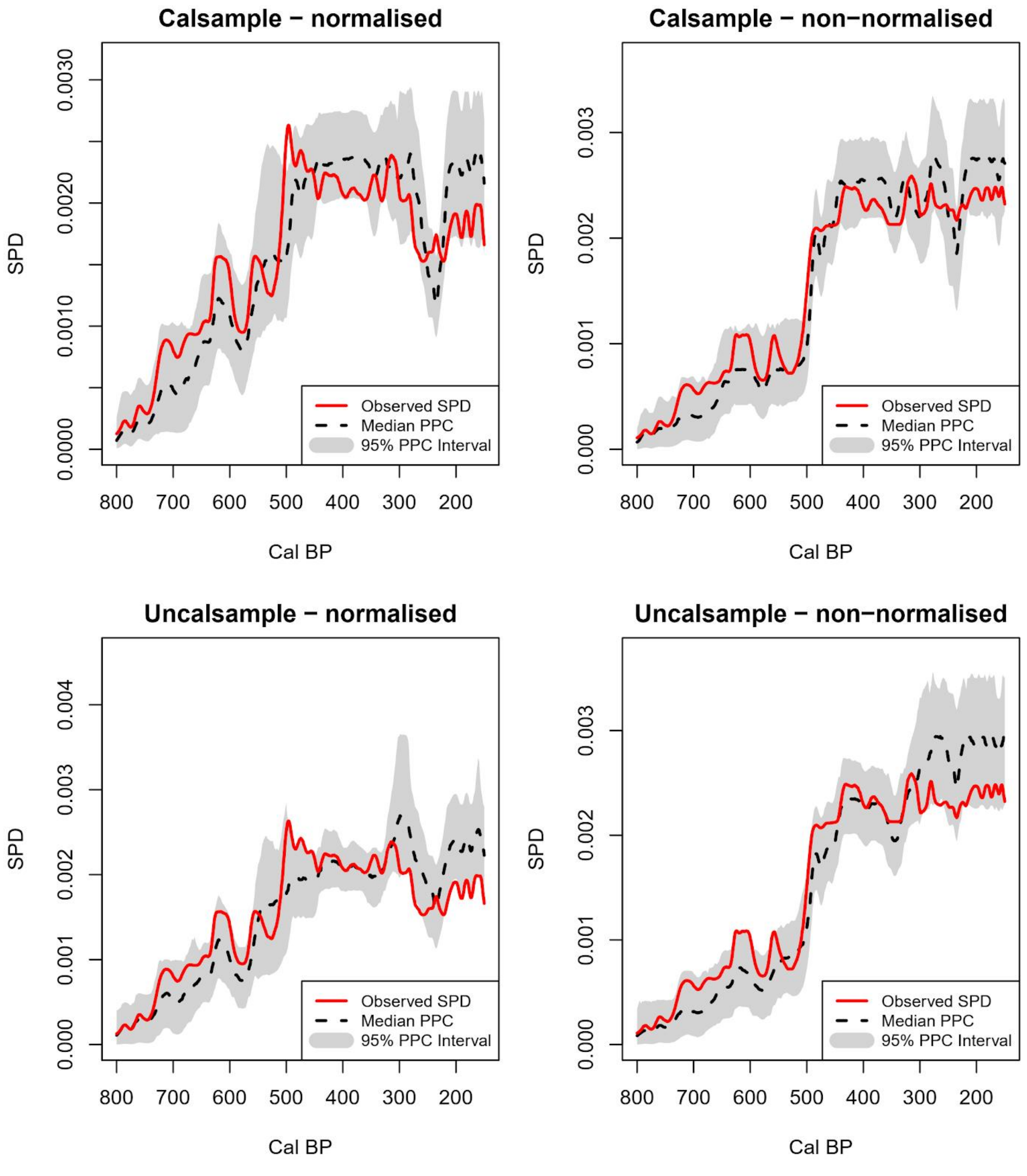

Figure S44. Posterior results for model 3 using four combinations of normalization and back-calibration. The solid red line is the observed SPD, the dashed black line is the median of the PCC, and the grey shading shows the 95% PPC interval.

Model 4

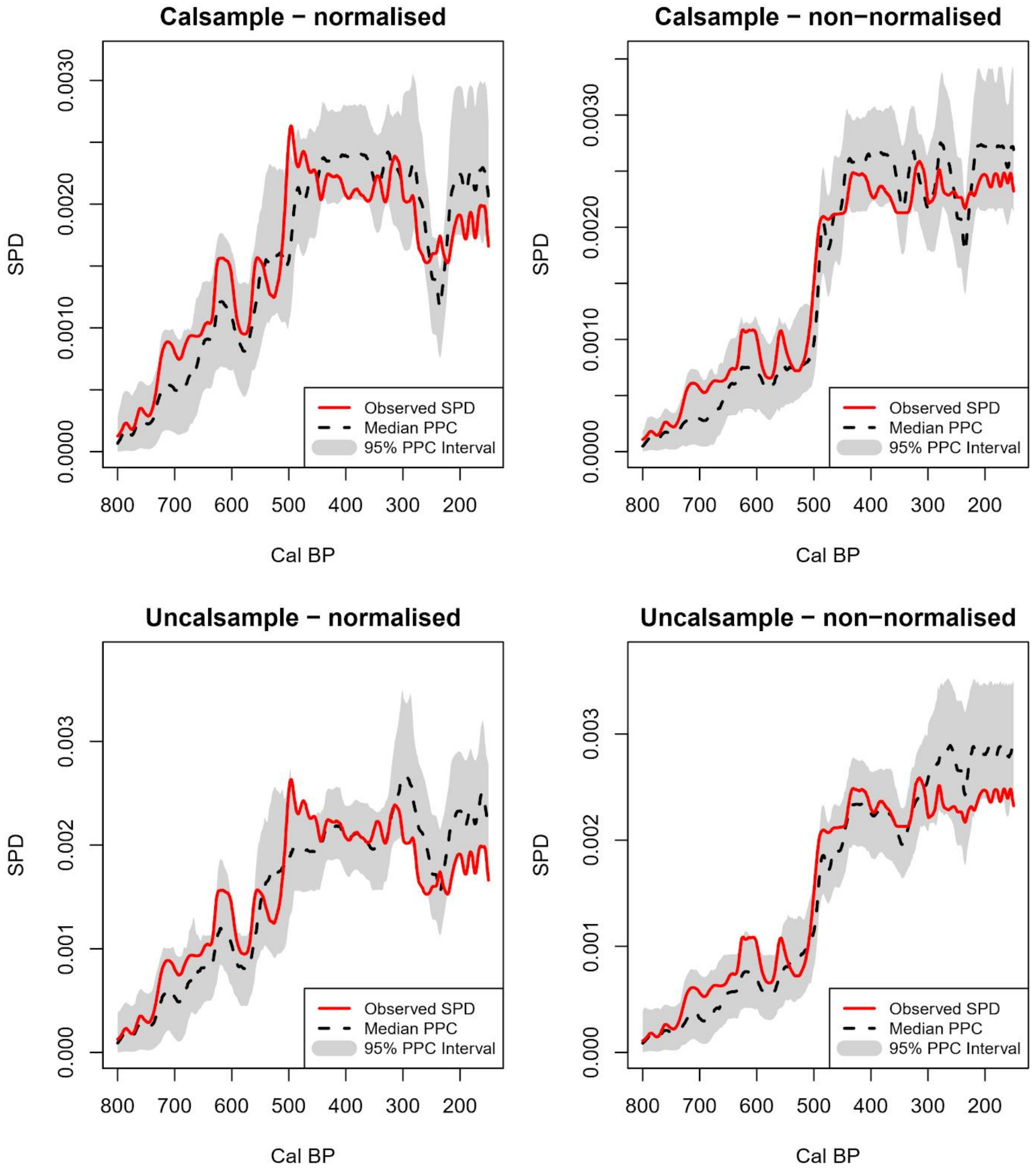

Figure S45. Posterior results for model 4 using four combinations of normalization and back-calibration. The solid red line is the observed SPD, the dashed black line is the median of the PCC, and the grey shading shows the 95% PPC interval.

Supplementary Note 11: Bayes Factor Matrices

The tables (S2-S9) below show the results of Bayes factor comparisons of model fits using both Euclidean distance and RMSE as the distance measure for the ABC rejection algorithm.

Bayes Factors using Euclidean distance

Table S2. Bayes factors using non-normalized dates and uncalsample. Note these are the values corresponding to the model results presented in the main text.

	Model 1	Model 2	Model 3	Model 4
Model 1	—	0.9471545	0.8759398	0.9137255
Model 2	1.055794	—	0.9248120	0.9647059
Model 3	1.141631	1.0813008	—	1.0431373
Model 4	1.094421	1.0365854	0.9586466	—

Table S3. Bayes factors using normalized dates and uncalsample.

	Model 1	Model 2	Model 3	Model 4
Model 1	—	0.9588477	0.8661710	0.9137255
Model 2	1.042918	—	0.9033457	0.9529412
Model 3	1.154506	1.1069959	—	1.0549020
Model 4	1.094421	1.0493827	0.9479554	—

Table S4. Bayes factors using normalized dates and calsample.

	Model 1	Model 2	Model 3	Model 4
Model 1	—	0.9186992	0.8692308	0.8432836
Model 2	1.088496	—	0.9461538	0.9179104
Model 3	1.150442	1.0569106	—	0.9701493
Model 4	1.185841	1.0894309	1.0307692	—

Table S5. Bayes factors using non-normalized dates and calsample.

	Model 1	Model 2	Model 3	Model 4
Model 1	—	0.8945148	0.8153846	0.7285223
Model 2	1.117925	—	0.9115385	0.8144330
Model 3	1.226415	1.0970464	—	0.8934708
Model 4	1.372642	1.2278481	1.1192308	—

Bayes Factors using normalized root mean squared error

Table S6. Bayes factors using normalized dates and uncalsample.

	Model 1	Model 2	Model 3	Model 4
Model 1	—	1.004237	0.8777778	0.9221790
Model 2	0.9957806	—	0.8740741	0.9182879
Model 3	1.1392405	1.144068	—	1.0505837
Model 4	1.0843882	1.088983	0.9518519	—

Table S7. Bayes factors using non-normalized dates and uncalsample.

	Model 1	Model 2	Model 3	Model 4
Model 1	—	0.9833333	0.8773234	0.9254902
Model 2	1.016949	—	0.8921933	0.9411765
Model 3	1.139831	1.1208333	—	1.0549020
Model 4	1.080508	1.0625000	0.9479554	—

Table S8. Bayes factors using normalized dates and calsample.

	Model 1	Model 2	Model 3	Model 4
Model 1	—	0.908	0.908	0.8315018
Model 2	1.101322	—	1.000	0.9157509
Model 3	1.101322	1.000	—	0.9157509
Model 4	1.202643	1.092	1.092	—

Table S9. Bayes factors using non-normalized dates and calsample.

	Model 1	Model 2	Model 3	Model 4
Model 1	—	0.9177489	0.7794118	0.7438596
Model 2	1.089623	—	0.8492647	0.8105263
Model 3	1.283019	1.1774892	—	0.9543860
Model 4	1.344340	1.2337662	1.0477941	—

Supplementary References

1. R Core Team. *R: A Language and Environment for Statistical Computing*. (R Foundation for Statistical Computing, 2020).
2. Crema, E. R. & Bevan, A. Inference from Large Sets of Radiocarbon Dates: Software and Methods. *Radiocarbon* **63**, 23–39 (2021).
3. Weninger, B., Clare, L., Jöris, O., Jung, R. & Edinborough, K. Quantum theory of radiocarbon calibration. *World Archaeology* **47**, 543–566 (2015).
4. Weninger, B. & Edinborough, K. Bayesian 14C-Rationality, Heisenberg uncertainty, and Fourier Transform: the Beauty of radiocarbon calibration. *Documenta Praehistorica* **47**, 536–559 (2020).
5. Lima, M. *et al.* Ecology of the collapse of Rapa Nui society. *Proceedings of the Royal Society B: Biological Sciences* **287**, 20200662 (2020).
6. Surovell, T. A., Byrd Finley, J., Smith, G. M., Brantingham, P. J. & Kelly, R. Correcting temporal frequency distributions for taphonomic bias. *Journal of Archaeological Science* **36**, 1715–1724 (2009).
7. Bevan, A. *et al.* Holocene fluctuations in human population demonstrate repeated links to food production and climate. *PNAS* **114**, E10524–E10531 (2017).
8. Hogg, A. G. *et al.* SHCal20 Southern Hemisphere Calibration, 0–55,000 Years cal BP. *Radiocarbon* **62**, 759–778 (2020).
