# Supplementary material for: Approximate Bayesian Computation of radiocarbon and paleoenvironmental record shows population resilience on Rapa Nui (Easter Island)
Source: Nat Commun. 2021 Jun 24;12:3939. doi: 10.1038/s41467-021-24252-z (PMC8225912; doi:10.1038/s41467-021-24252-z)
Supplement: Supplementary file 2 — Reporting Summary [file 41467_2021_24252_MOESM2_ESM.pdf]

nature research

Corresponding author(s): Robert J. DiNapoli

Last updated by author(s): Jun 1, 2021

Reporting Summary

Nature Research wishes to improve the reproducibility of the work that we publish. This form provides structure for consistency and transparency in reporting. For further information on Nature Research policies, see our Editorial Policies and the Editorial Policy Checklist.

Statistics

For all statistical analyses, confirm that the following items are present in the figure legend, table legend, main text, or Methods section.

n/a	Confirmed	
☐	☒	The exact sample size (n) for each experimental group/condition, given as a discrete number and unit of measurement
☒	☐	A statement on whether measurements were taken from distinct samples or whether the same sample was measured repeatedly
☒	☐	The statistical test(s) used AND whether they are one- or two-sided<br>*Only common tests should be described solely by name; describe more complex techniques in the Methods section.*
☐	☒	A description of all covariates tested
☒	☐	A description of any assumptions or corrections, such as tests of normality and adjustment for multiple comparisons
☐	☒	A full description of the statistical parameters including central tendency (e.g. means) or other basic estimates (e.g. regression coefficient) AND variation (e.g. standard deviation) or associated estimates of uncertainty (e.g. confidence intervals)
☒	☐	For null hypothesis testing, the test statistic (e.g. F, t, r) with confidence intervals, effect sizes, degrees of freedom and P value noted<br>*Give P values as exact values whenever suitable.*
☐	☒	For Bayesian analysis, information on the choice of priors and Markov chain Monte Carlo settings
☐	☒	For hierarchical and complex designs, identification of the appropriate level for tests and full reporting of outcomes
☒	☐	Estimates of effect sizes (e.g. Cohen's d, Pearson's r), indicating how they were calculated

Our web collection on statistics for biologists contains articles on many of the points above.

Software and code

Policy information about availability of computer code

Data collection	Data was collected from previously published sources using Microsoft Excel.
Data analysis	We conducted all of our analyses in R version 4.0.3 (R Core Team 2020) using the rcarbon 1.4.1 package (Crema and Bevan 2021). Fully reproducible code can be found at https://github.com/rdinapoli/RN_demography

For manuscripts utilizing custom algorithms or software that are central to the research but not yet described in published literature, software must be made available to editors and reviewers. We strongly encourage code deposition in a community repository (e.g. GitHub). See the Nature Research guidelines for submitting code & software for further information.

Data

Policy information about availability of data

All manuscripts must include a data availability statement. This statement should provide the following information, where applicable:

- Accession codes, unique identifiers, or web links for publicly available datasets
- A list of figures that have associated raw data
- A description of any restrictions on data availability

All data necessary to reproduce these analyses are available at https://github.com/rdinapoli/RN_demography

Field-specific reporting

Ecological, evolutionary & environmental sciences study design

All studies must disclose on these points even when the disclosure is negative.

Study description	This study is a quantitative analysis of radiocarbon dates and paleoenvironmental data that uses Approximate Bayesian Computation for paleodemogaphic modeling.
Research sample	The sample is a collection of radiocarbon dates from archaeological contexts on Rapa Nui and paleoecological data from the island. These data were obtained from previously published sources, and references to these sources are fully described in the main text and SI.
Sampling strategy	Our sample is composed of all radiocarbon dates from the island that come from unambiguous archaeological contexts with a clear contextual association with ancient settlements.
Data collection	Our data is a compilation of data from published sources, as detailed in the main text and SI.
Timing and spatial scale	Since the inception of radiocarbon dating on the island until the timing of submission. The spatial scale is the island of Rapa Nui.
Data exclusions	We excluded radiocarbon samples classified as Class 3 by Mulrooney (2013), which do not meet current chronometric hygiene criteria, and samples not from settlement sites.
Reproducibility	The code necessary to reproduce the results of each component of the analysis is available at https://github.com/rdinapoli/RN_demography. In all model runs we set a random seed to ensure reproducibility.
Randomization	Not applicable.
Blinding	Not applicable.

Did the study involve field work? ☐ Yes ☒ No

Reporting for specific materials, systems and methods

We require information from authors about some types of materials, experimental systems and methods used in many studies. Here, indicate whether each material, system or method listed is relevant to your study. If you are not sure if a list item applies to your research, read the appropriate section before selecting a response.

Materials & experimental systems

n/a	Involved in the study
☒	☐ Antibodies
☒	☐ Eukaryotic cell lines
☒	☐ Palaeontology and archaeology
☒	☐ Animals and other organisms
☒	☐ Human research participants
☒	☐ Clinical data
☒	☐ Dual use research of concern

Methods

n/a	Involved in the study
☒	☐ ChIP-seq
☒	☐ Flow cytometry
☒	☐ MRI-based neuroimaging
